# Supplementary material for: New Amino Acid Schiff Bases as Anticancer Agents via Potential Mitochondrial Complex I-Associated Hexokinase Inhibition and Targeting AMP-Protein Kinases/mTOR Signaling Pathway
Source: Molecules. 2021 Sep 2;26(17):5332. doi: 10.3390/molecules26175332 (PMC8434356; doi:10.3390/molecules26175332)
Supplement: Supplementary file 1 [file molecules-26-05332-s001.zip › molecules-1356805-supplementary.pdf]

molecules

MDPI

Supplementary Materials

New amino acid Schiff bases as anticancer agents via potential mitochondrial complex I-associated hexokinase inhibition and targeting AMPK/mTOR signaling pathway

Ahmed A. Noser[1,] Aboubakr H. Abdelmonsef[2]* and Maha M. Salem[3]

[1]Organic Chemistry, Chemistry Department, Faculty of Science, Tanta University, Tanta 31527, Egypt

[2]*Chemistry Department, Faculty of Science, South Valley University, Qena 83523, Egypt

[3]Biochemistry Division, Chemistry Department, Faculty of Science, Tanta University, Tanta 31527, Egypt

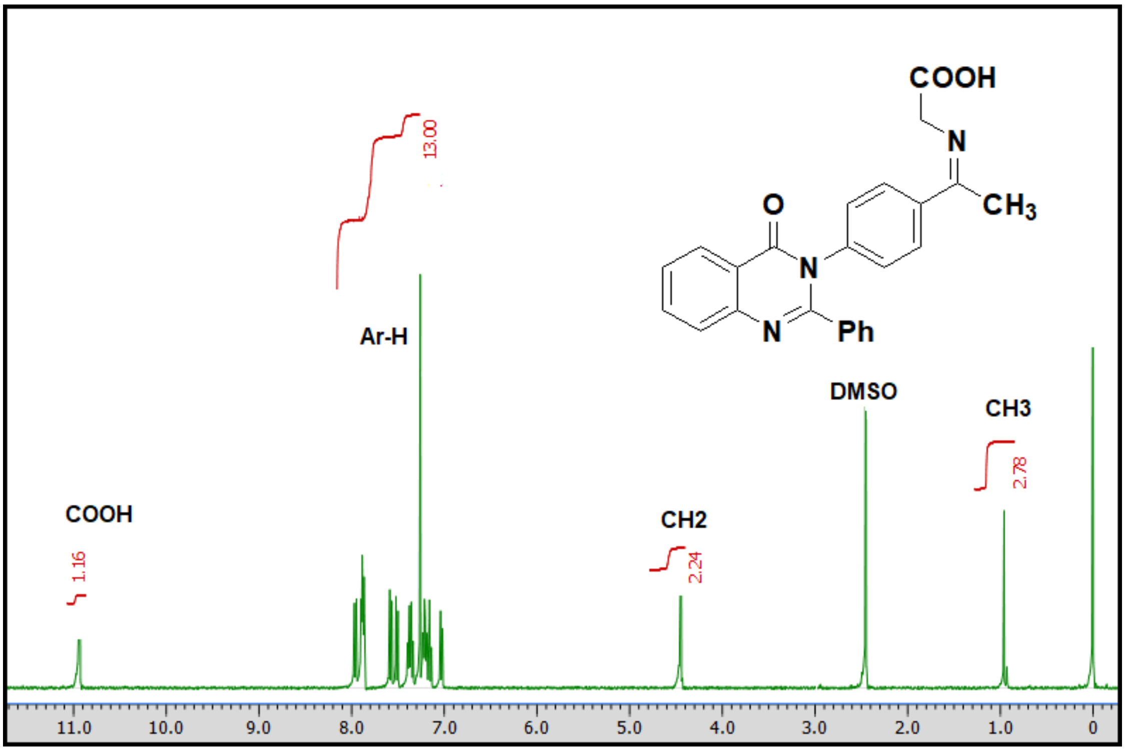

Figure S1. ^{1}H-NMR of compound 3

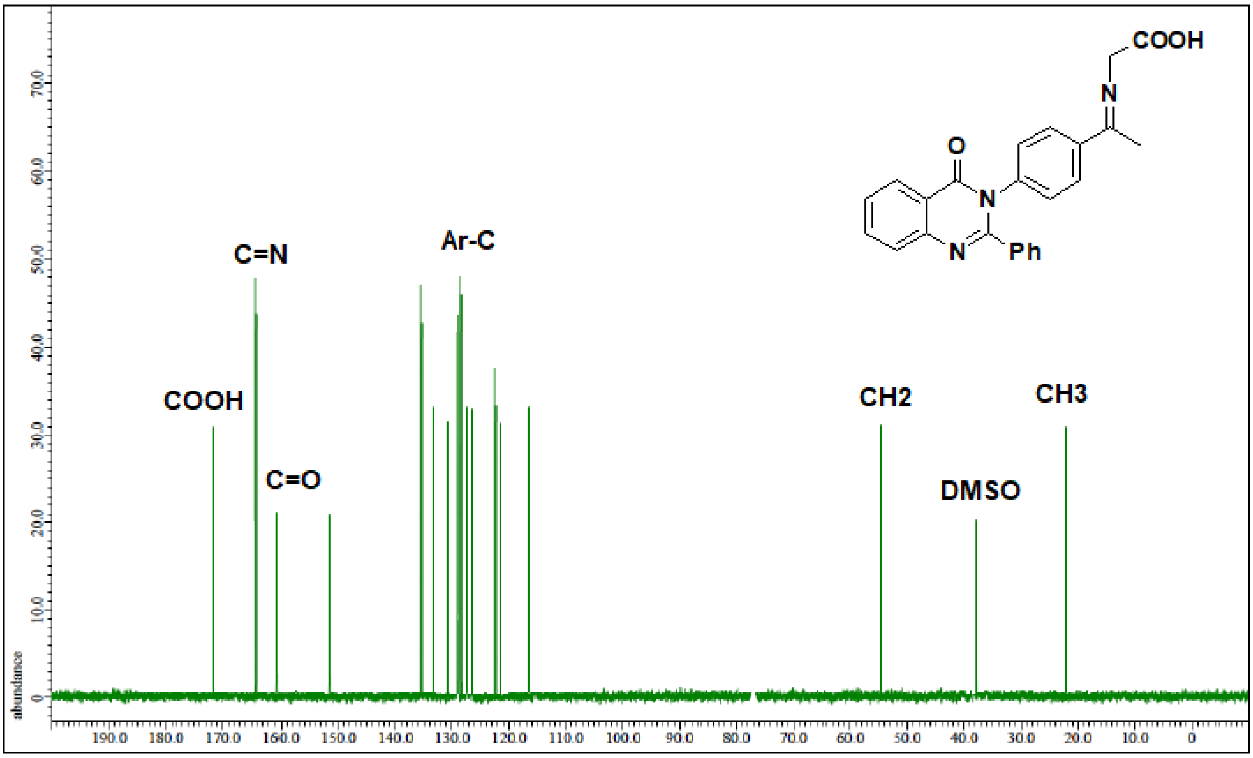

Figure S2. ^{13}C-NMR of compound 3

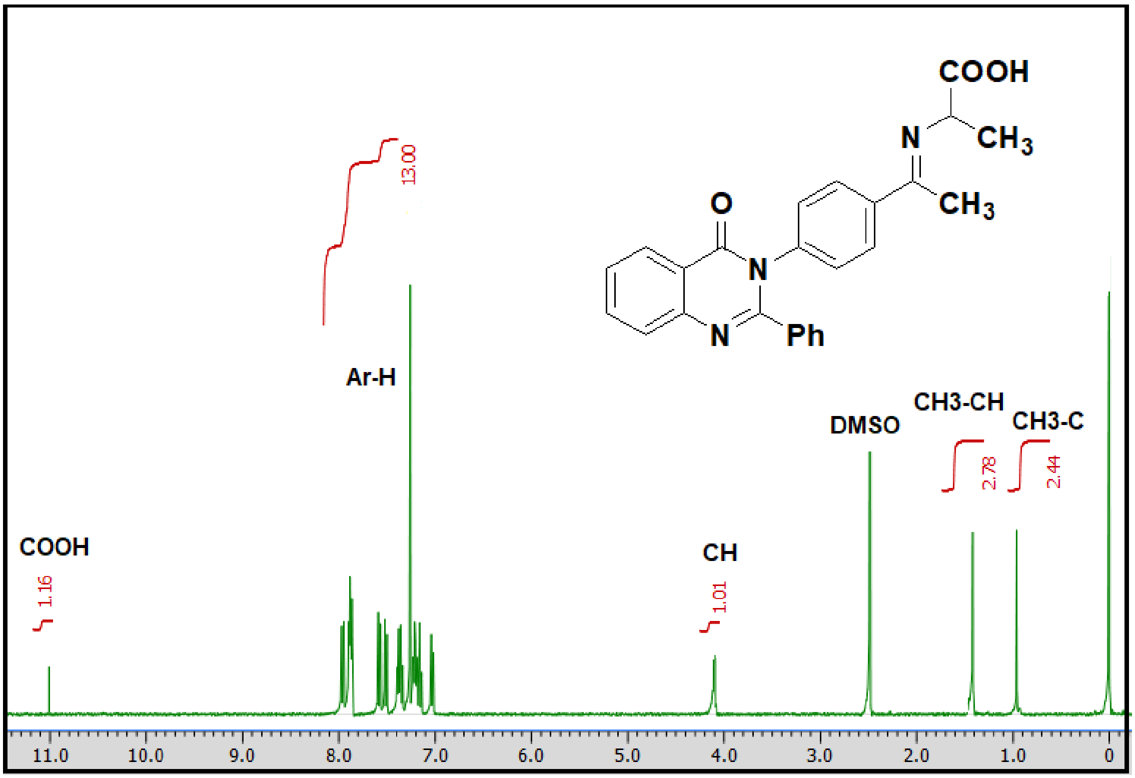

Figure S3. ^{1}H-NMR of compound 4

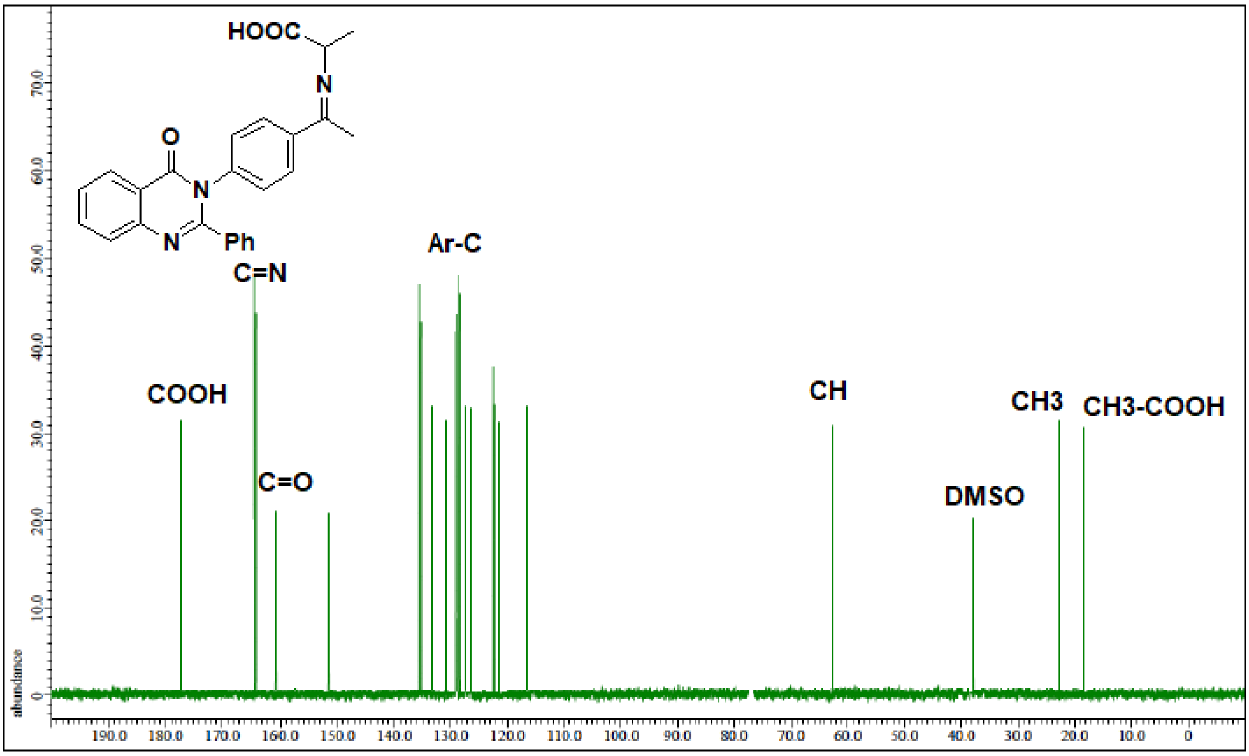

Figure S4. ^{13}C-NMR of compound 4

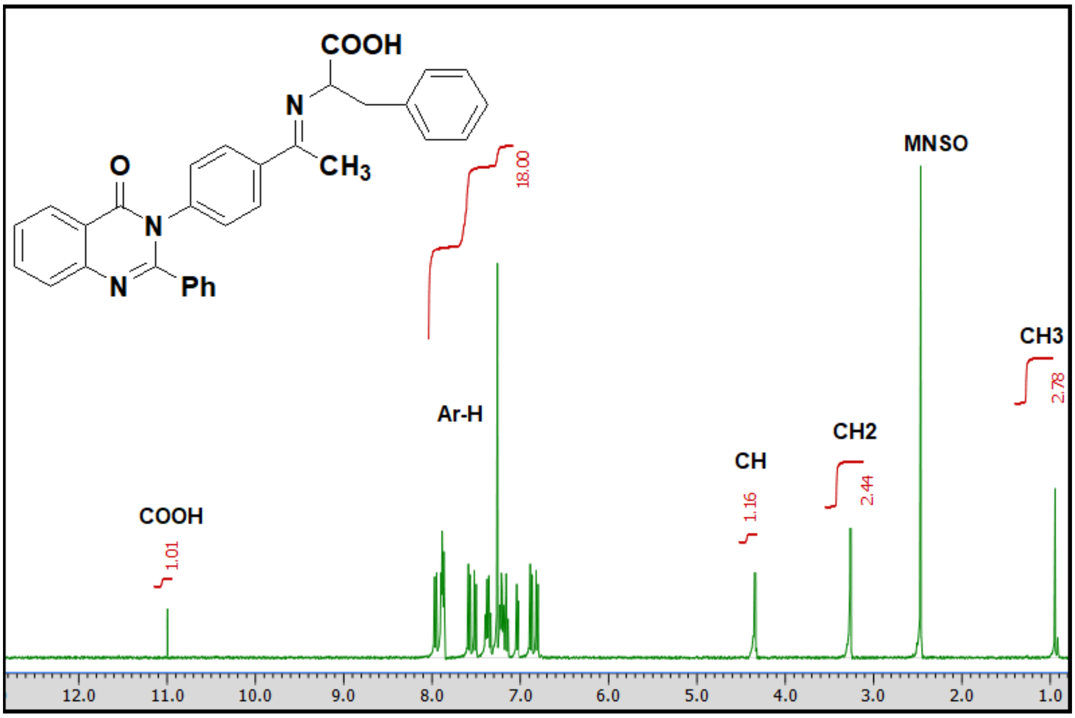

Figure S5. ^{1}H-NMR of compound 5

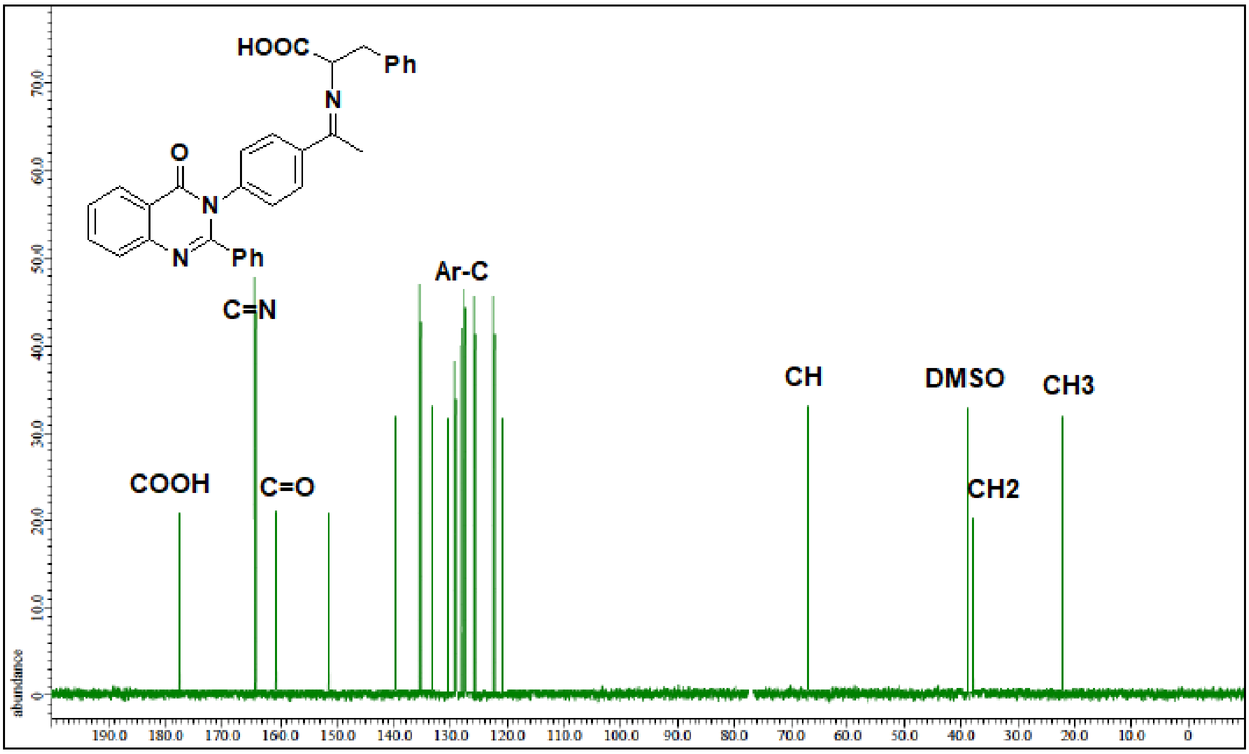

Figure S6. ^{13}C-NMR of compound 5

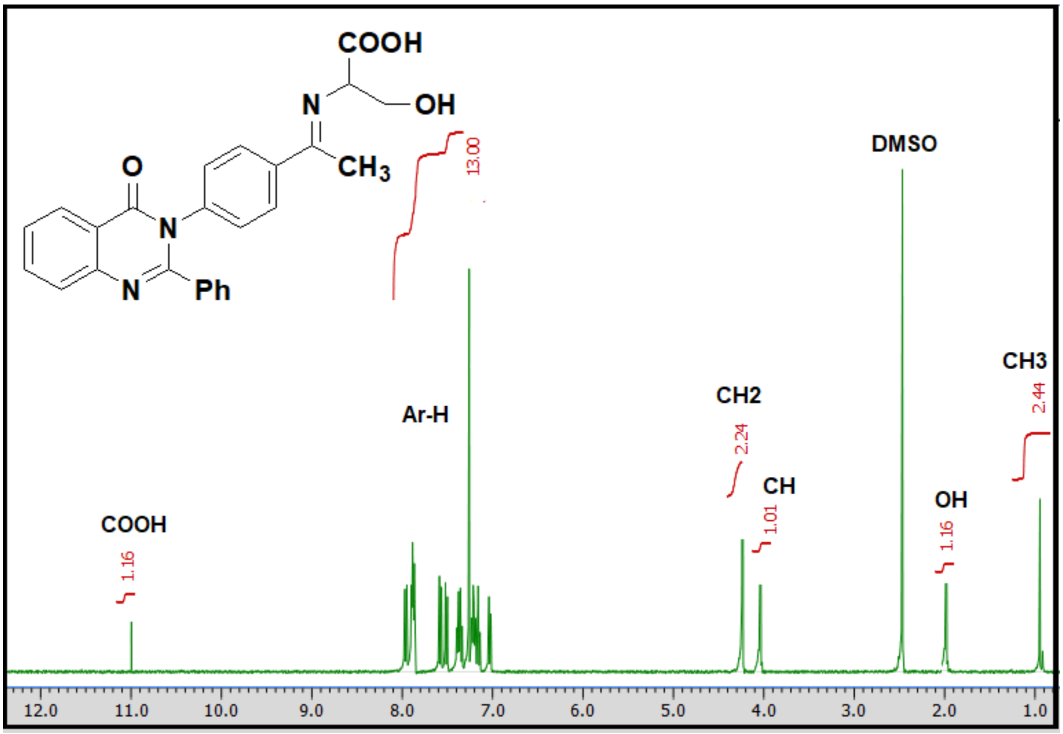

Figure S7. ^{1}H-NMR of compound 6

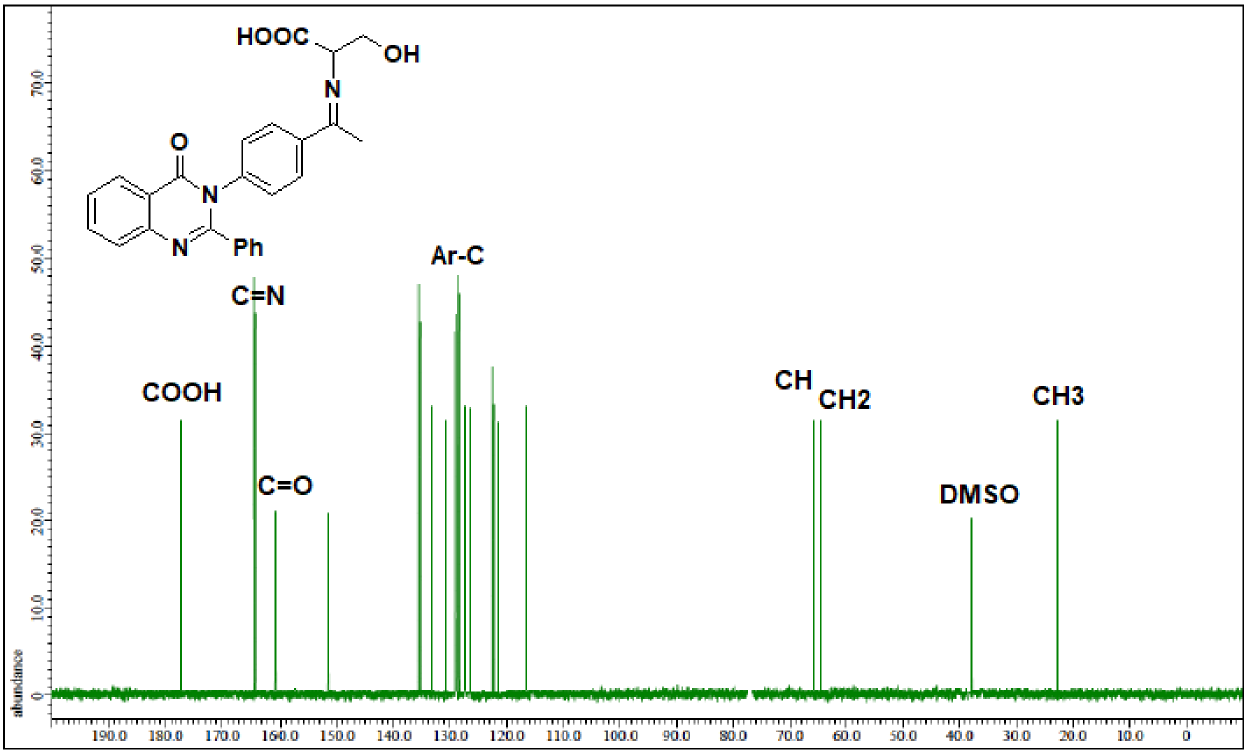

Figure S8. ^{13}C-NMR of compound 6

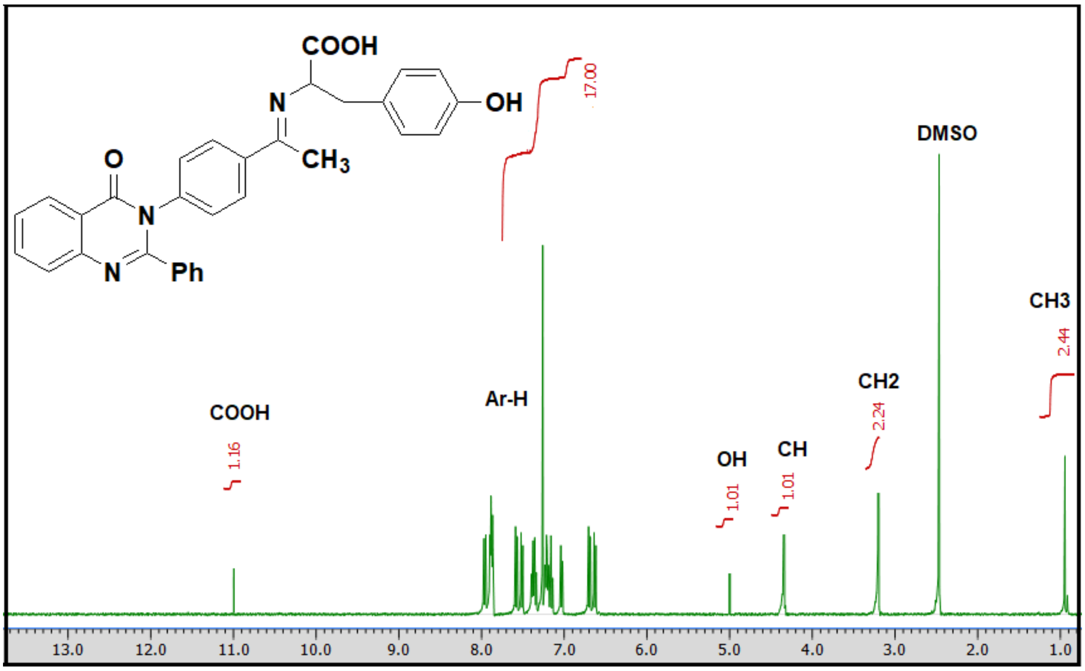

Figure S9. ^{1}H-NMR of compound 7

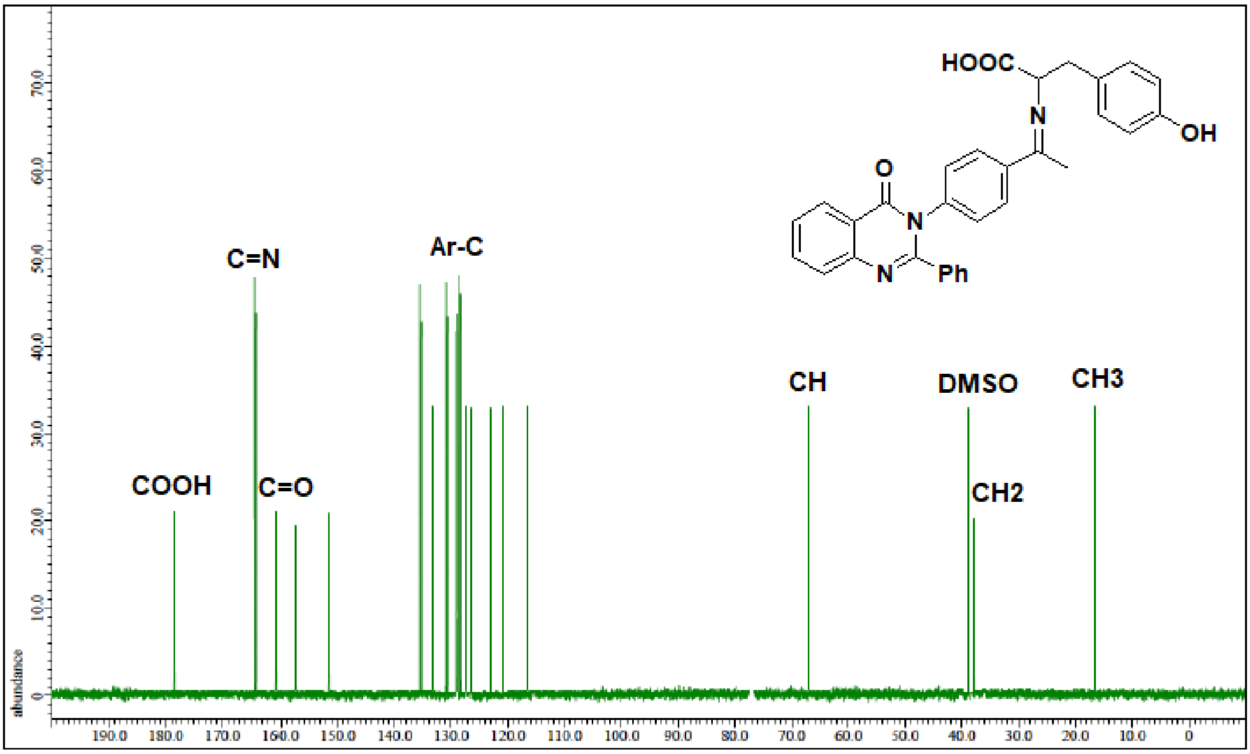

Figure S10. ^{13}C-NMR of compound 7

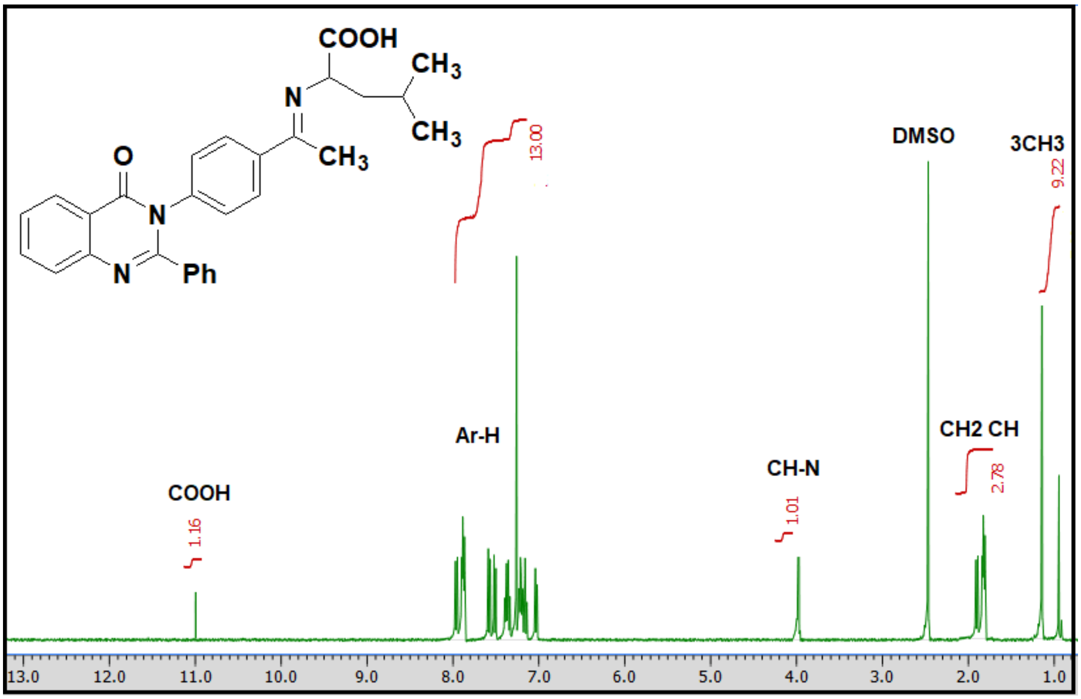

Figure S11. ^{1}H-NMR of compound 8

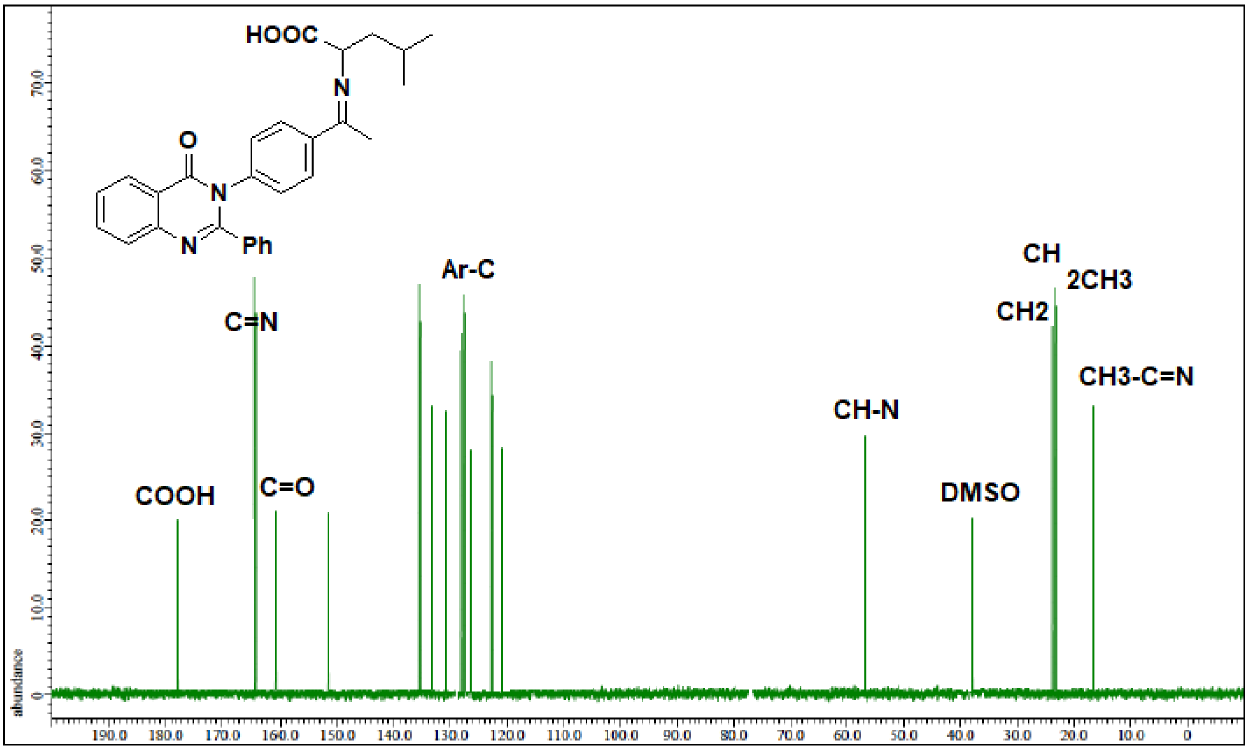

Figure S12. ^{13}C-NMR of compound 8

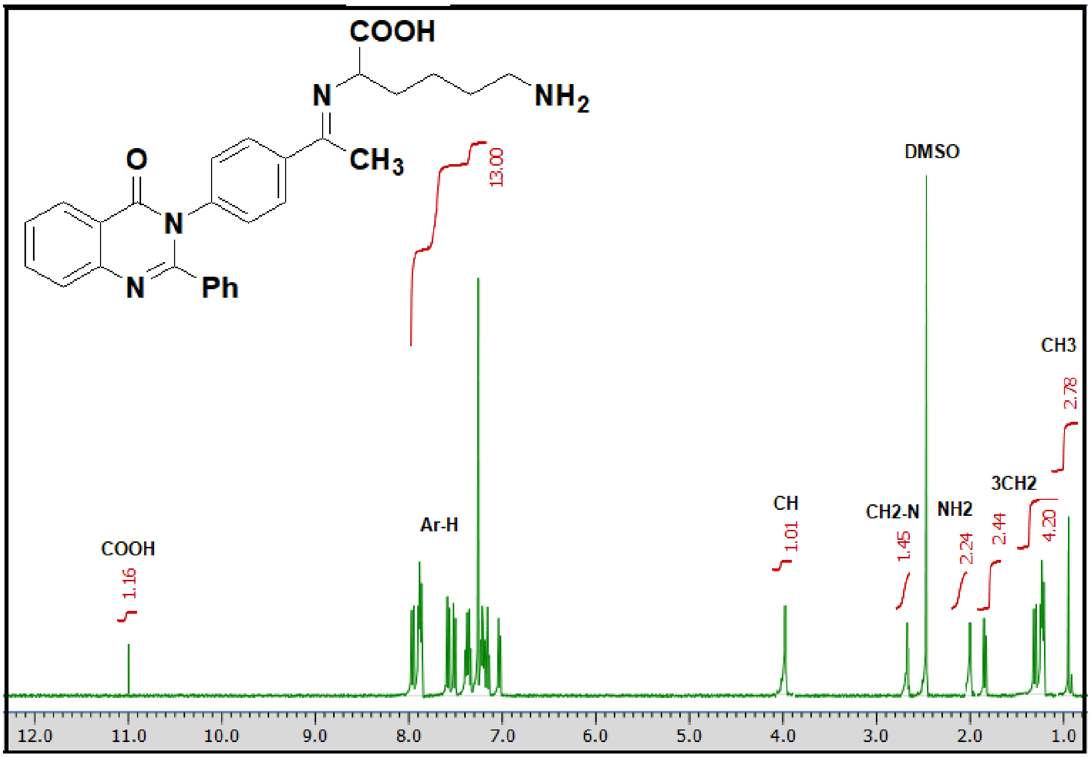

Figure S13. ^{1}H-NMR of compound 9

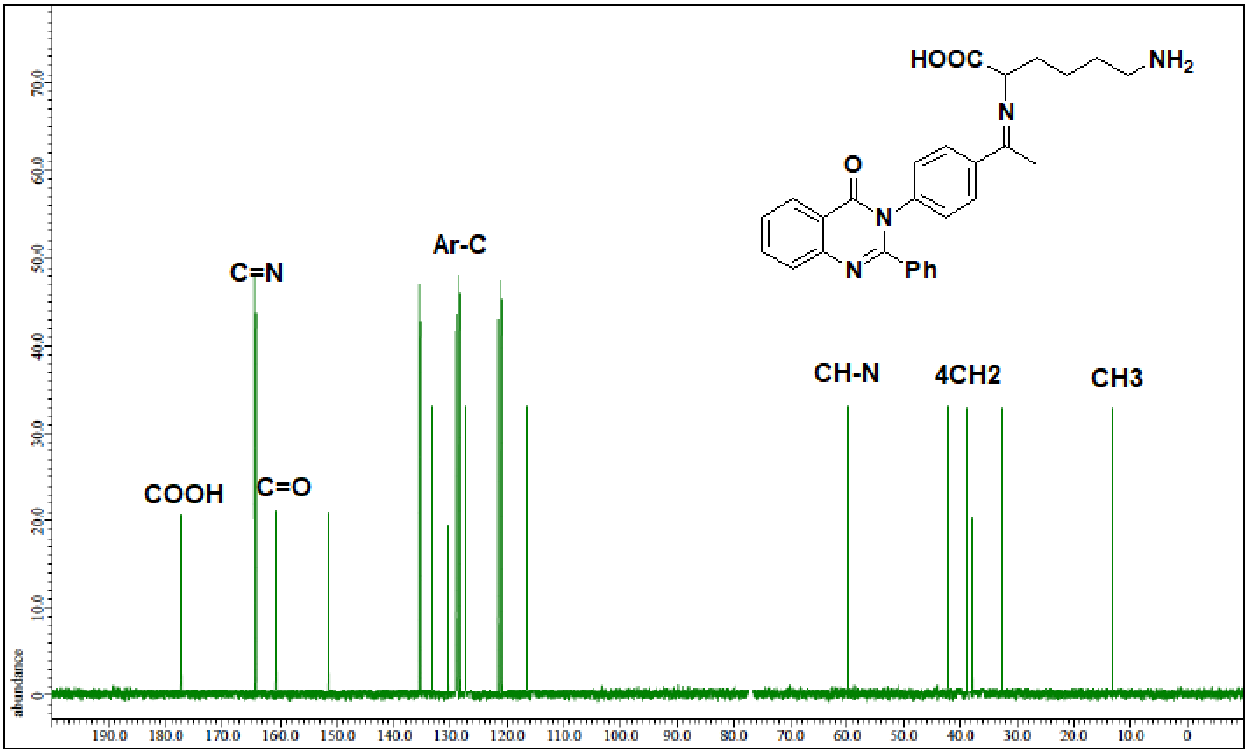

Figure S14. ^{13}C-NMR of compound 9

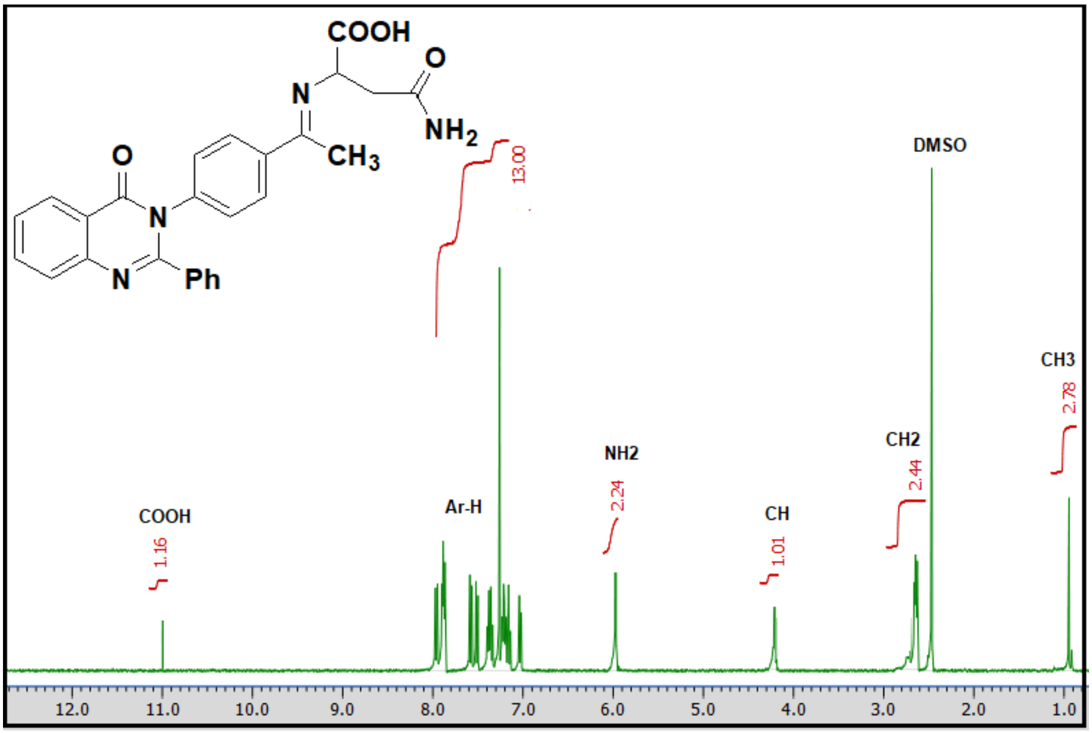

Figure S15. ^{1}H-NMR of compound 10

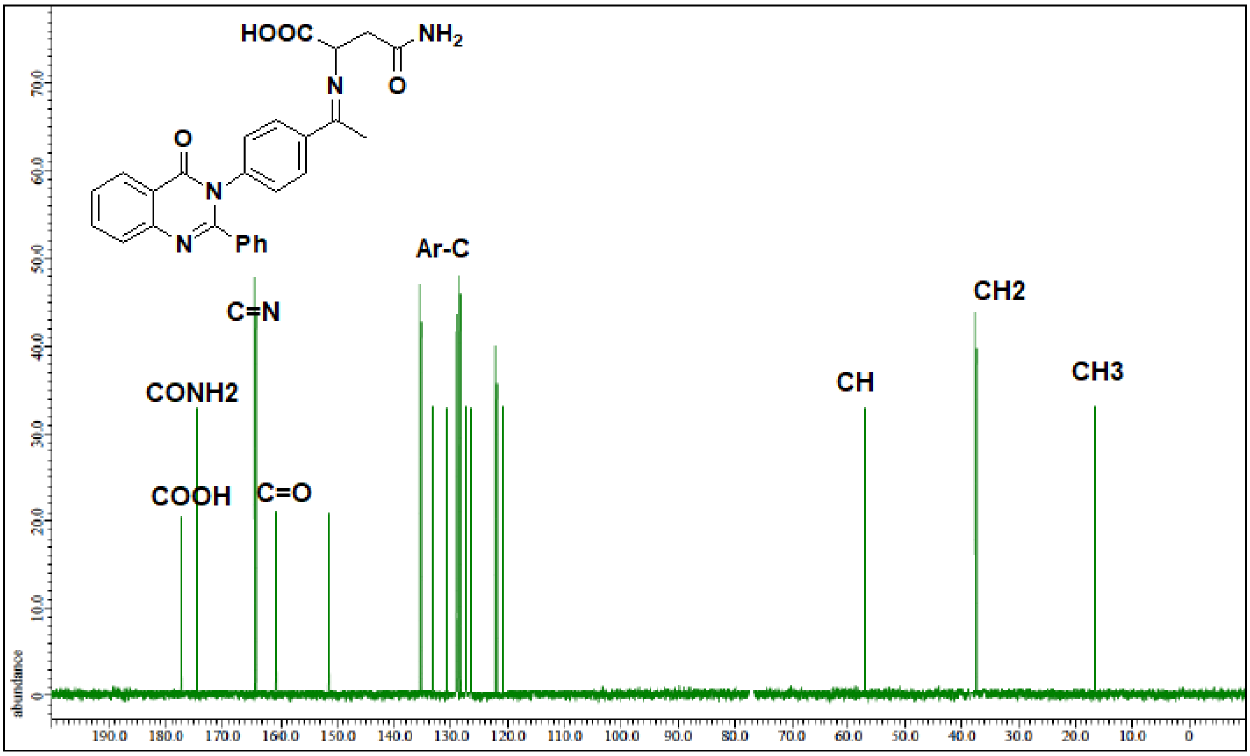

Figure S16. ^{13}C-NMR of compound 10

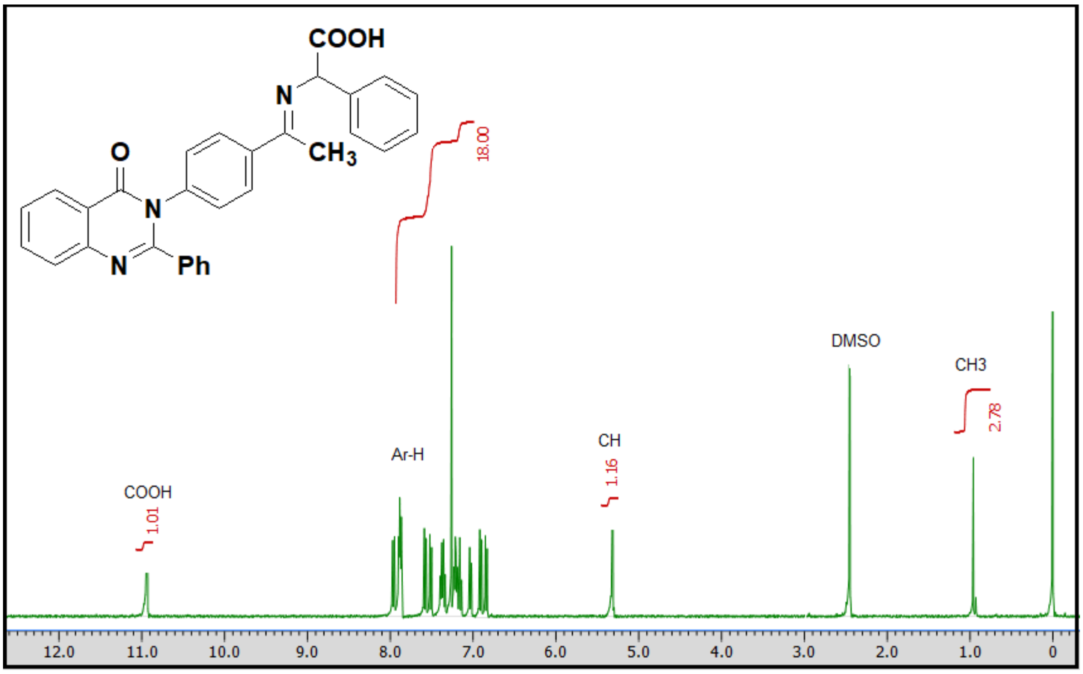

Figure S17. ^{1}H-NMR of compound 11

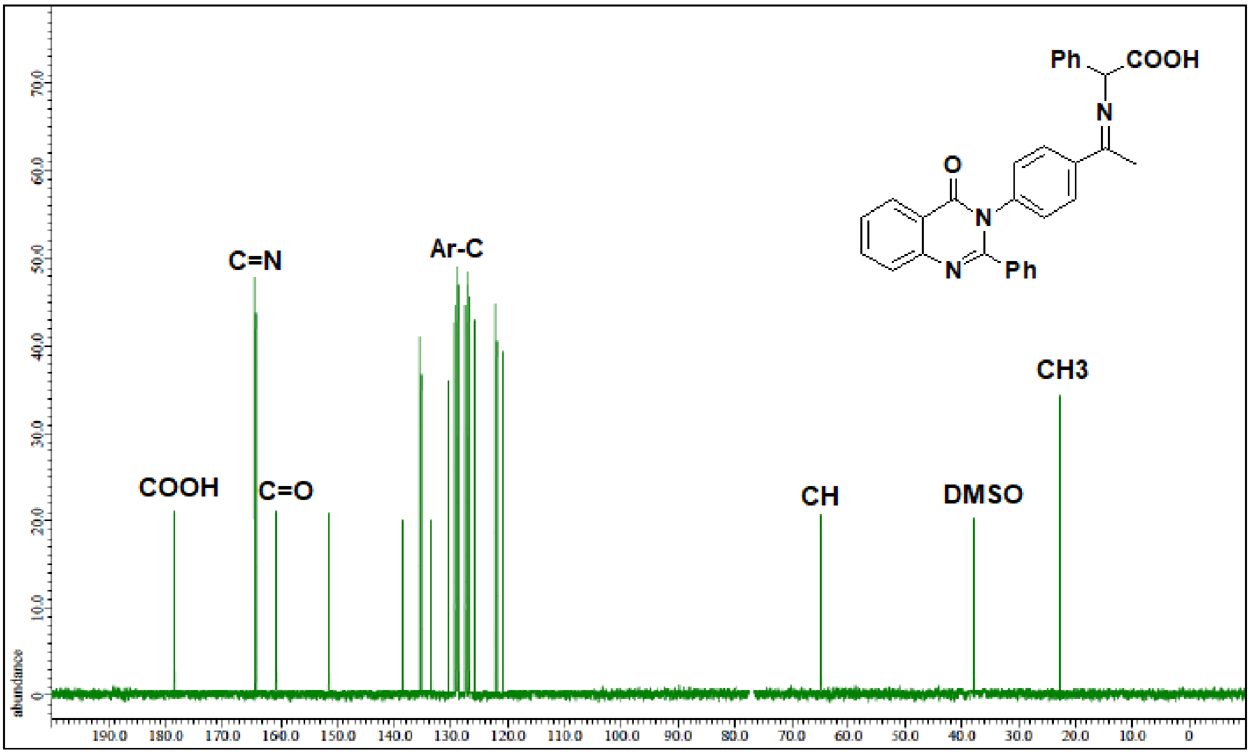

Figure S18. ^{13}C-NMR of compound 11

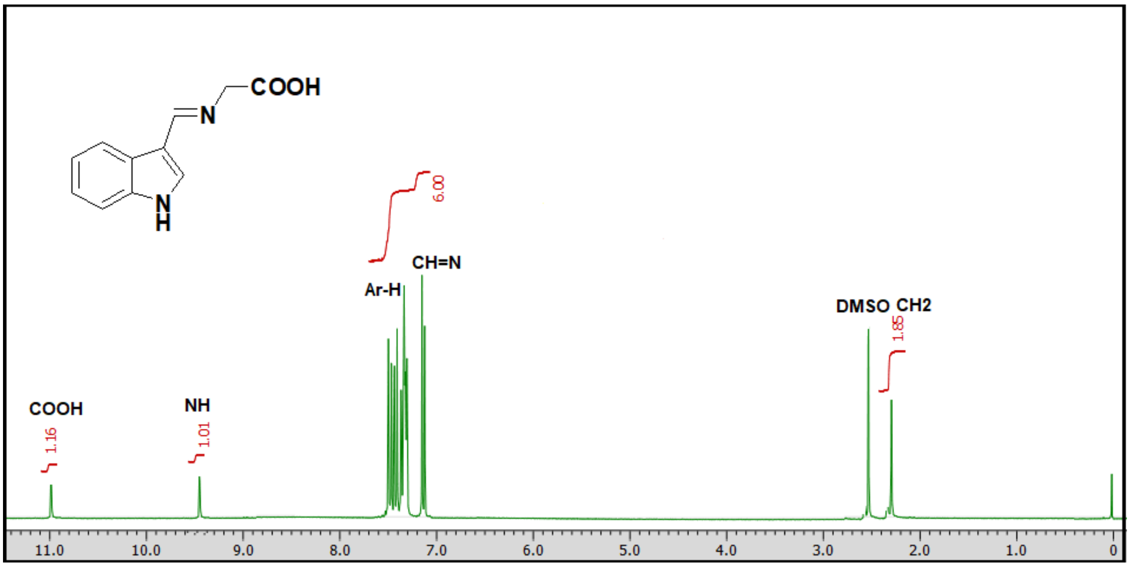

Figure S19. ^{1}H-NMR of compound 12

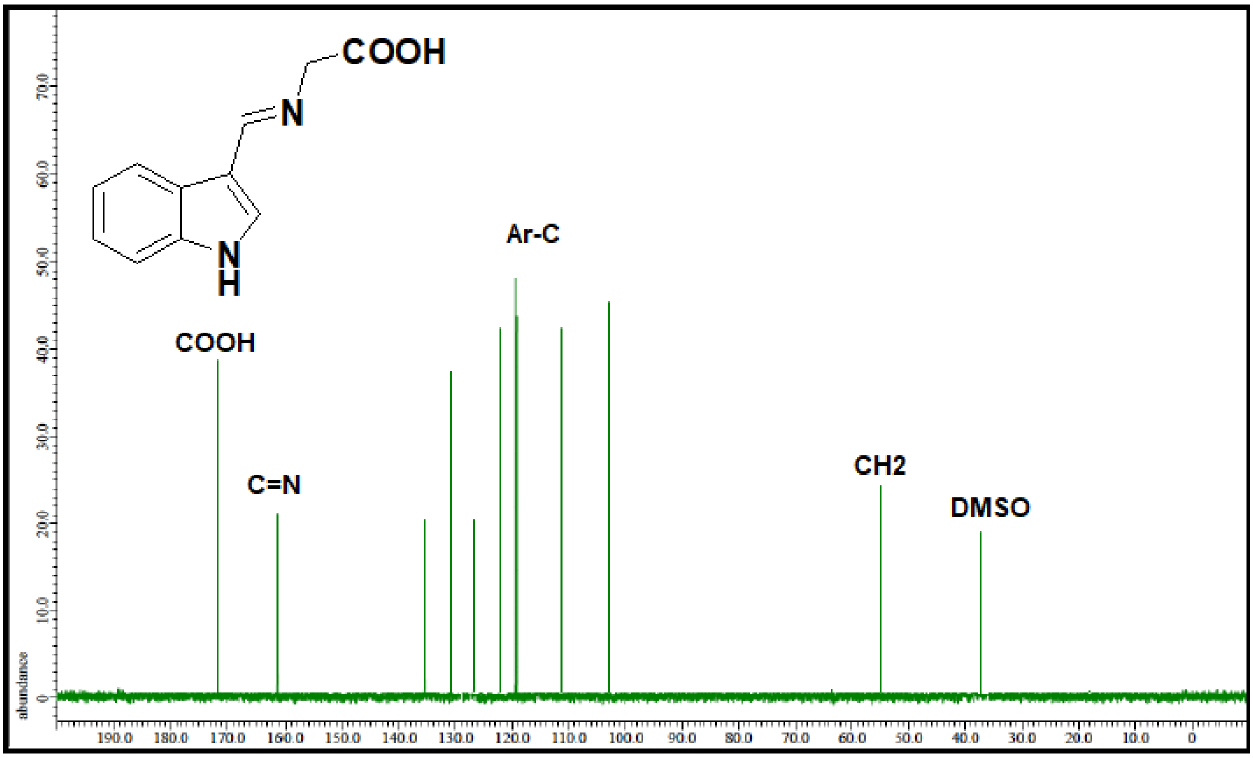

Figure S20. ^{13}C-NMR of compound 12

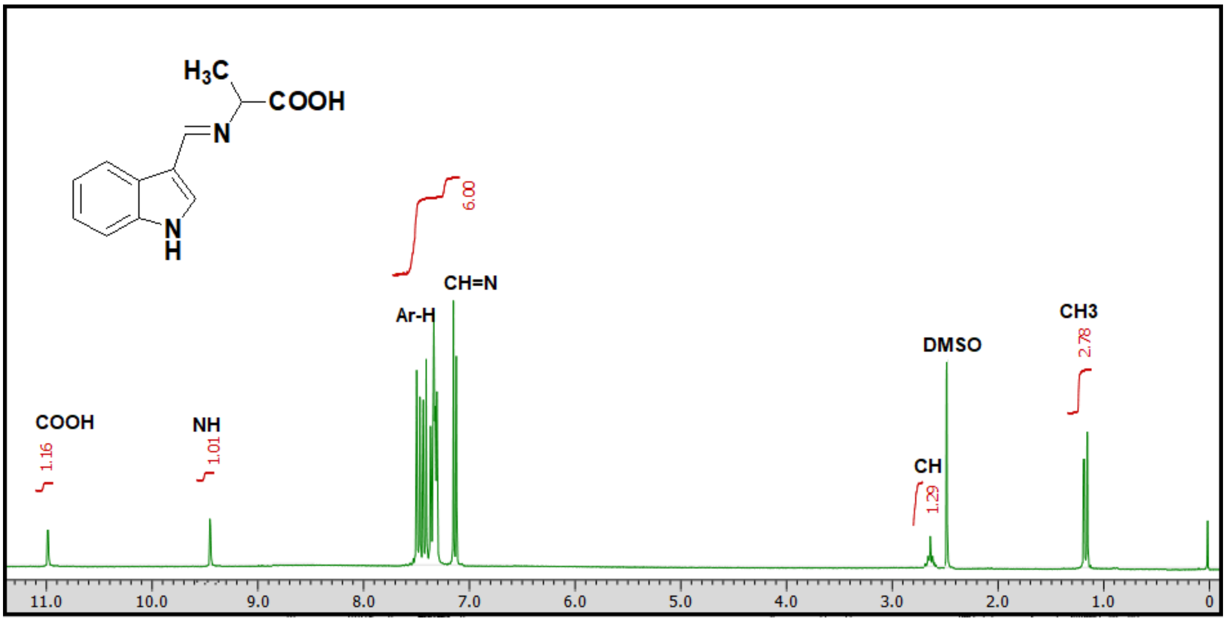

Figure S21. ^{1}H-NMR of compound 13

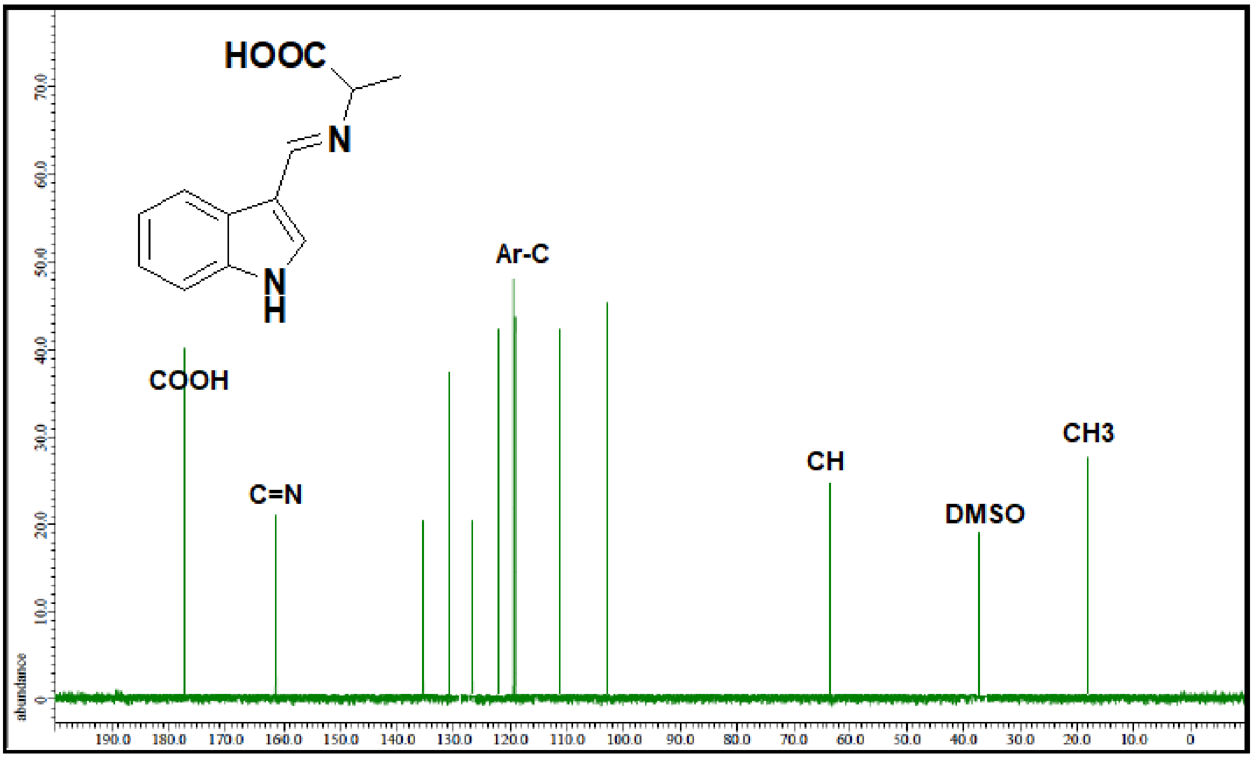

Figure S22. ^{13}C-NMR of compound 13

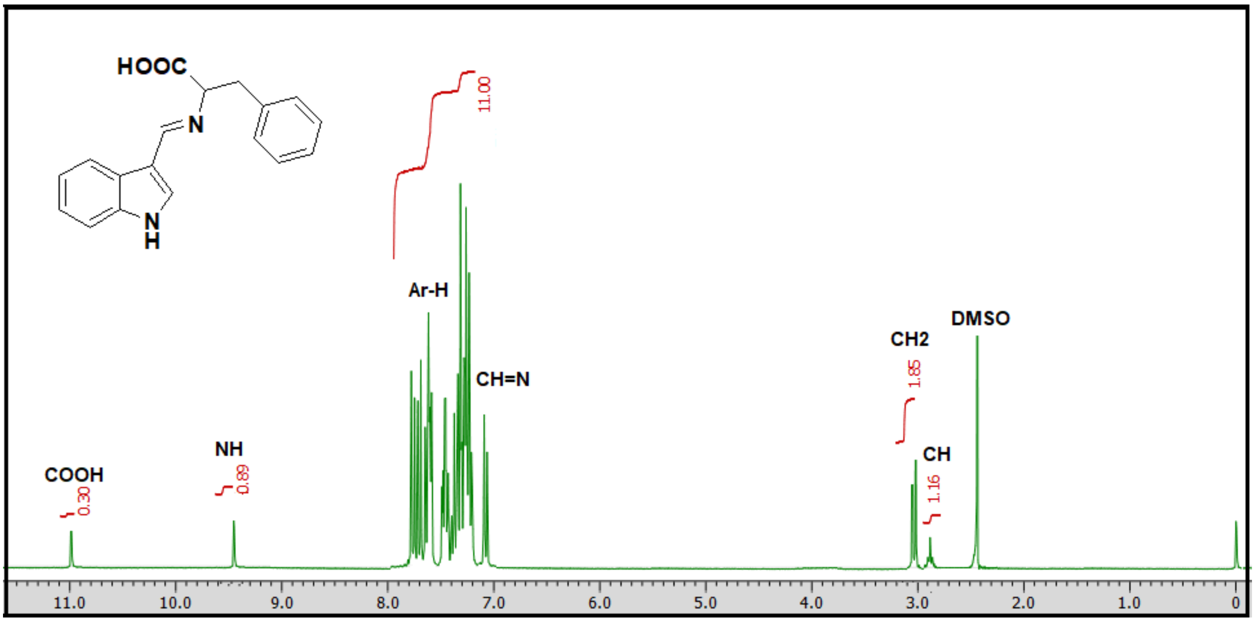

Figure S23. ^{1}H-NMR of compound 14

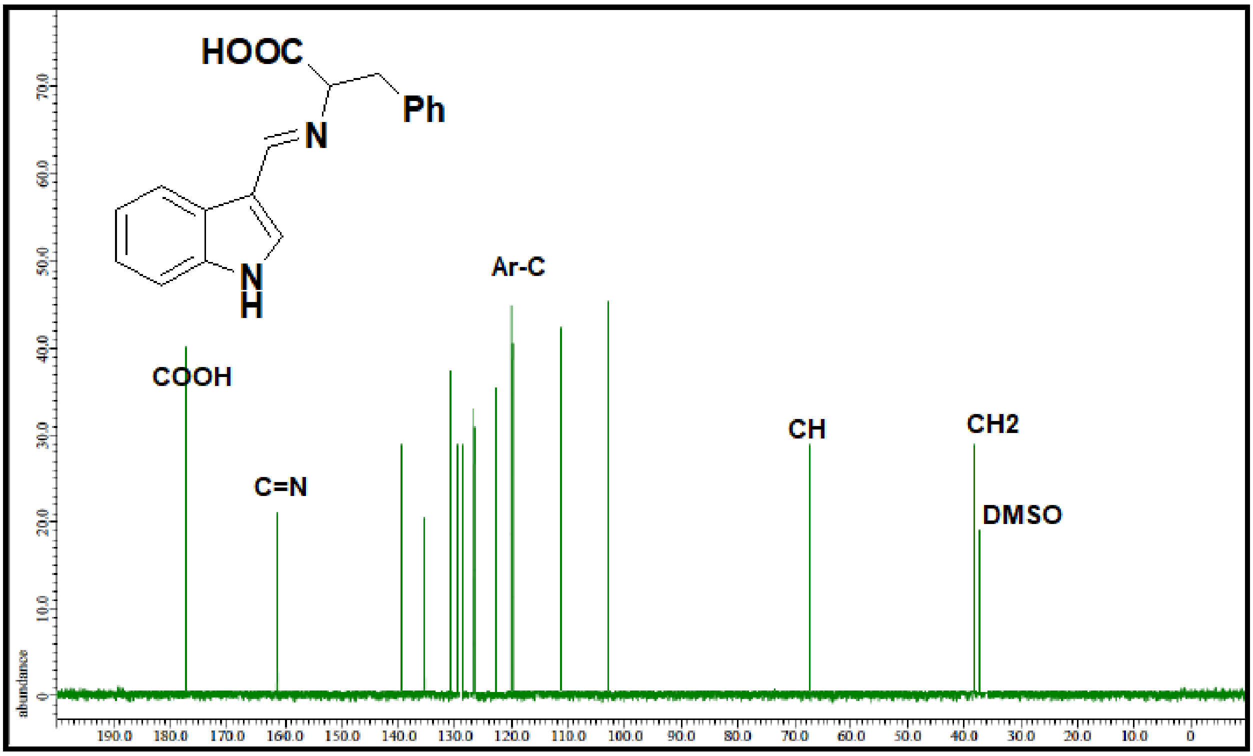

Figure S24. ^{13}C-NMR of compound 14

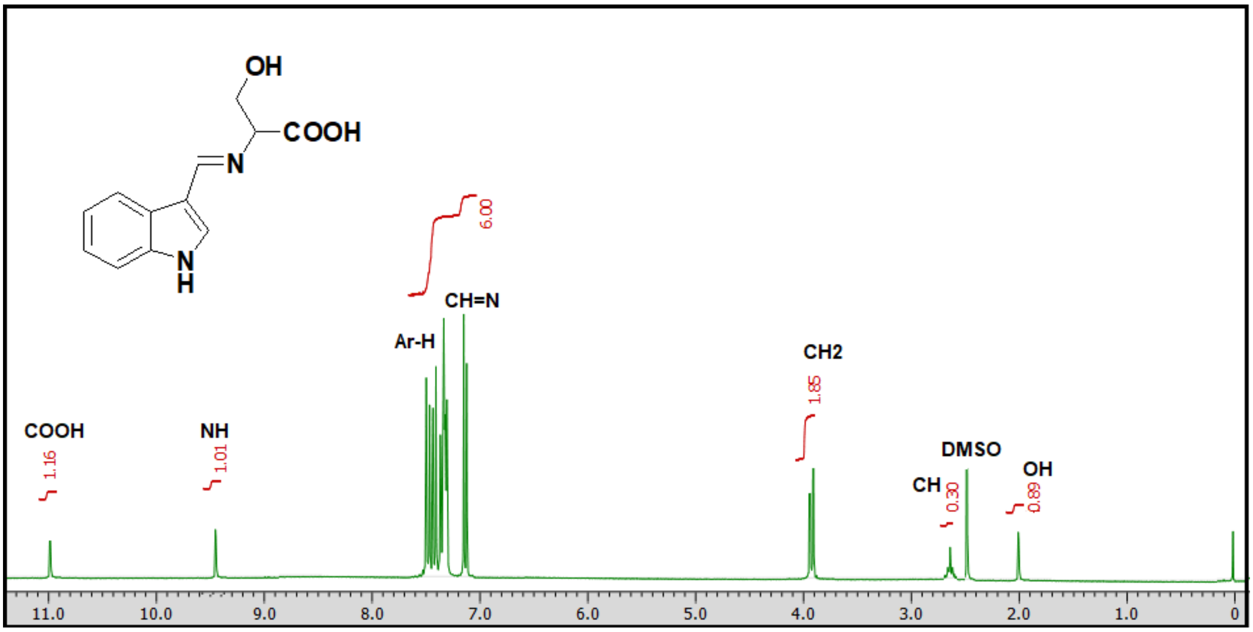

Figure S25. ^{1}H-NMR of compound 15

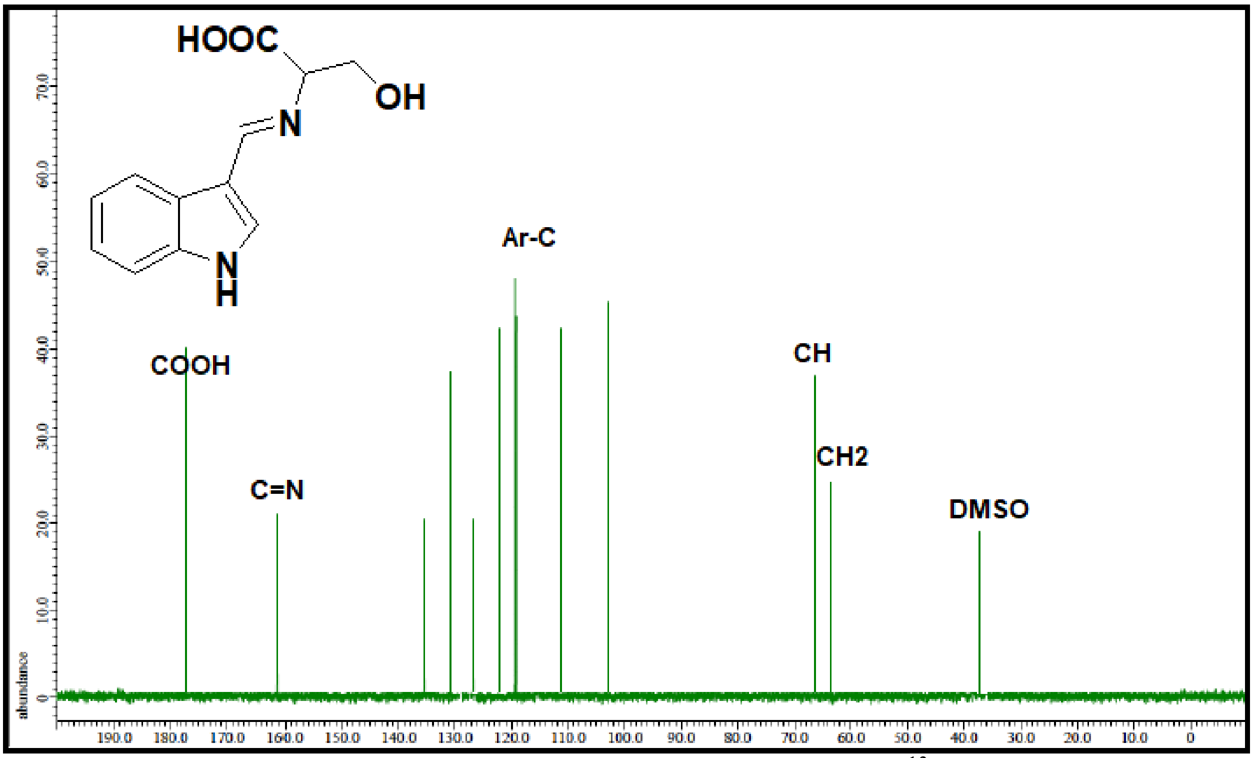

Figure S26. ^{13}C-NMR of compound 15

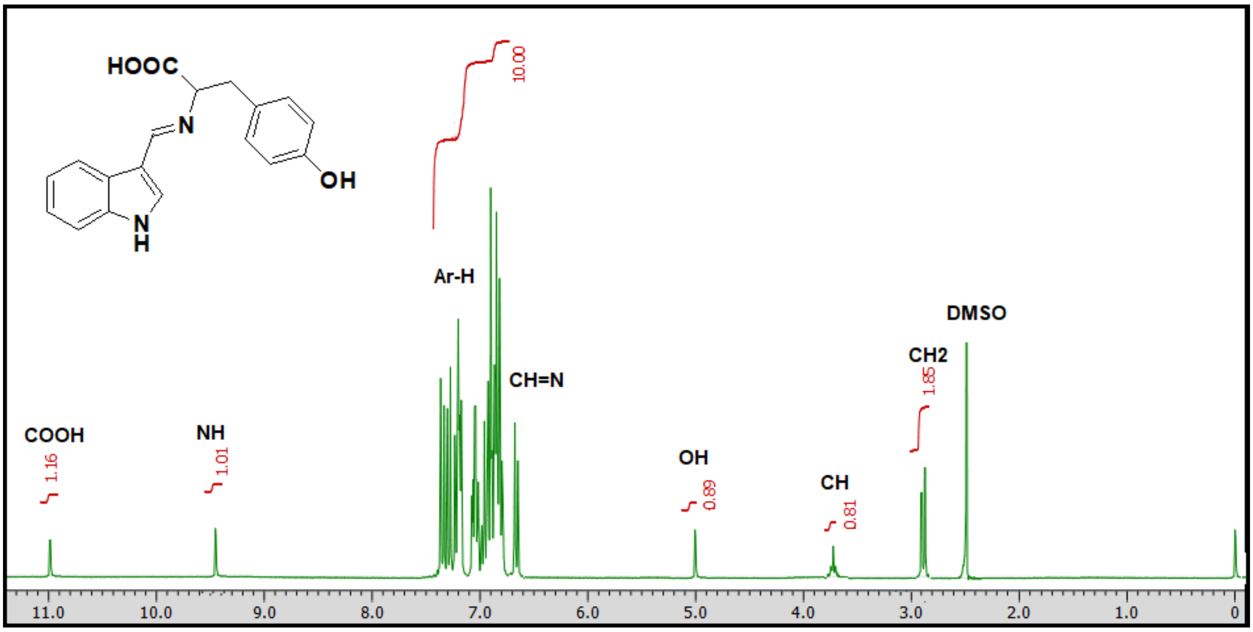

Figure S27. ^{1}H-NMR of compound 16

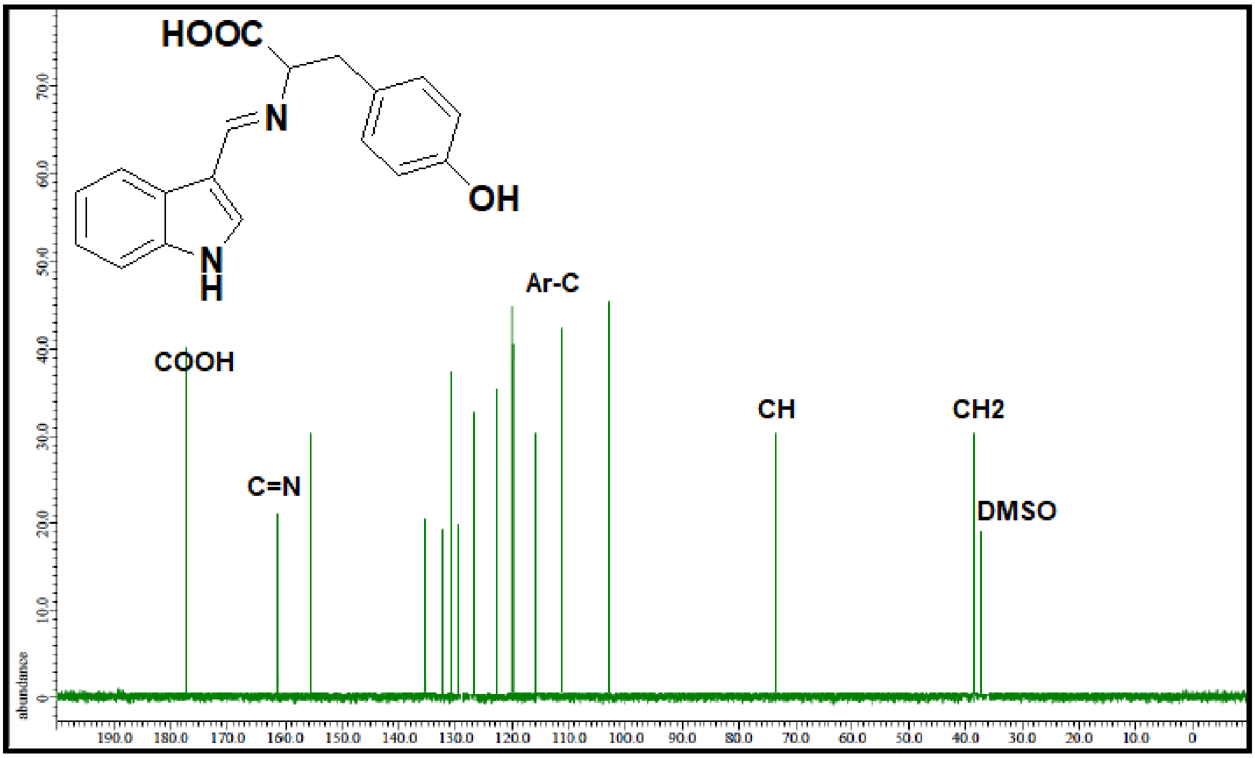

Figure S28. ^{13}C-NMR of compound 16

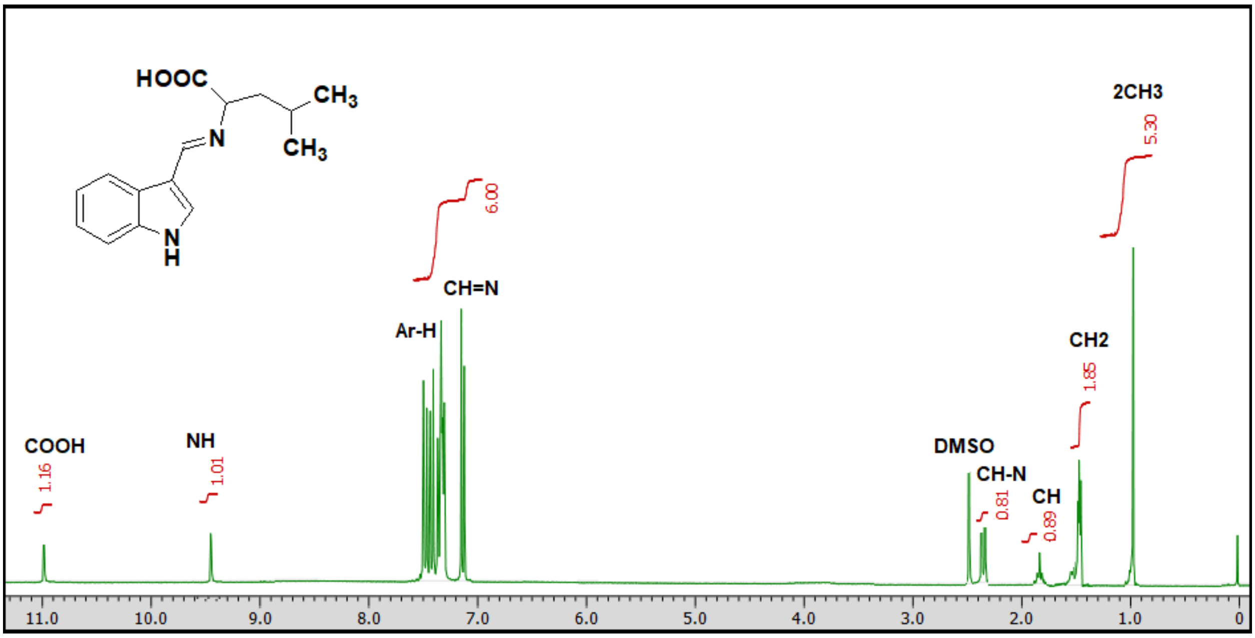

Figure S29. ^{1}H-NMR of compound 17

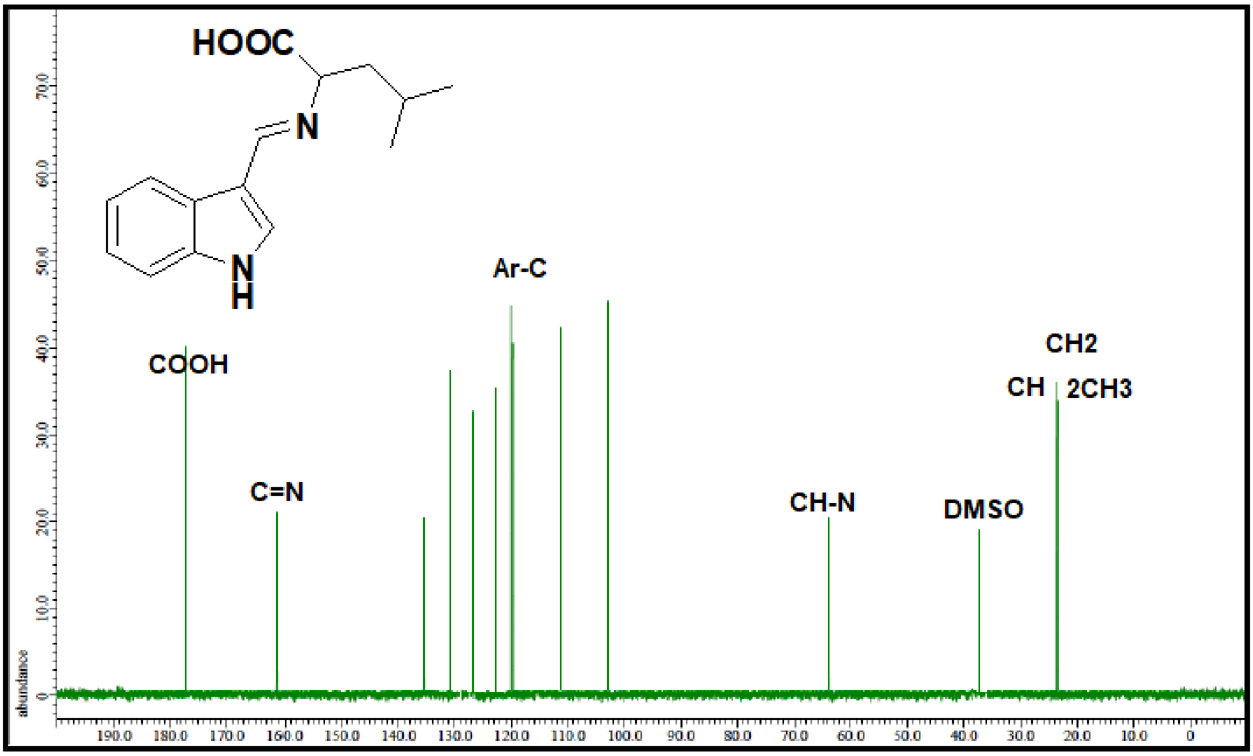

Figure S30. ^{13}C-NMR of compound 17

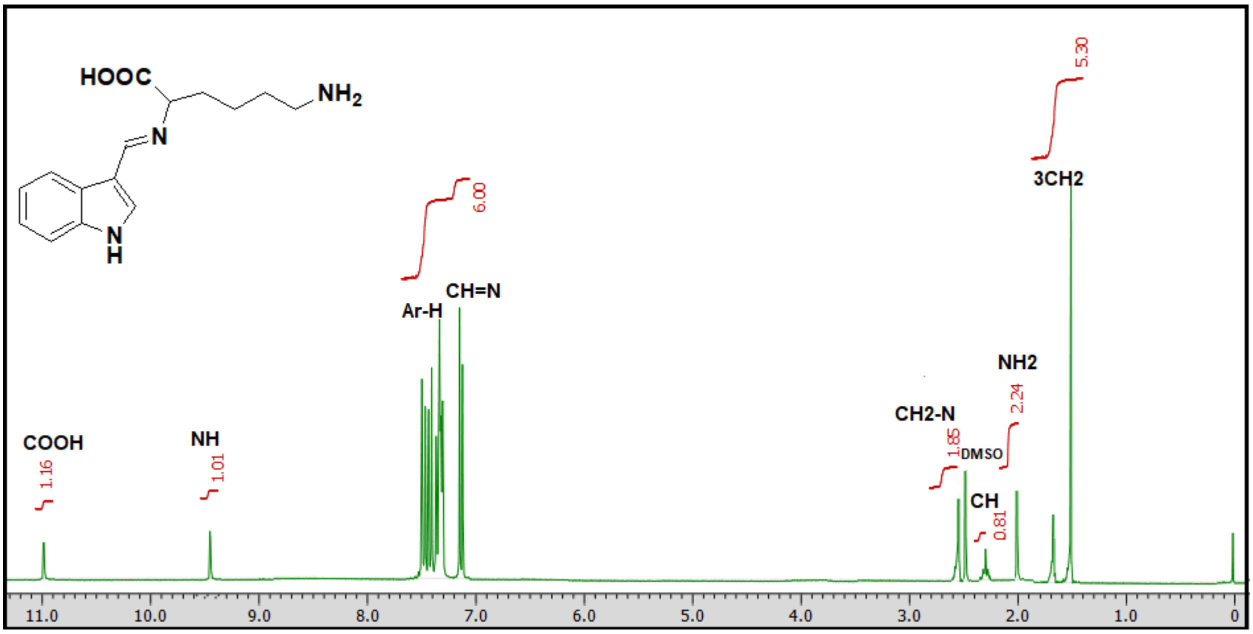

Figure S31. ^{1}H-NMR of compound 18

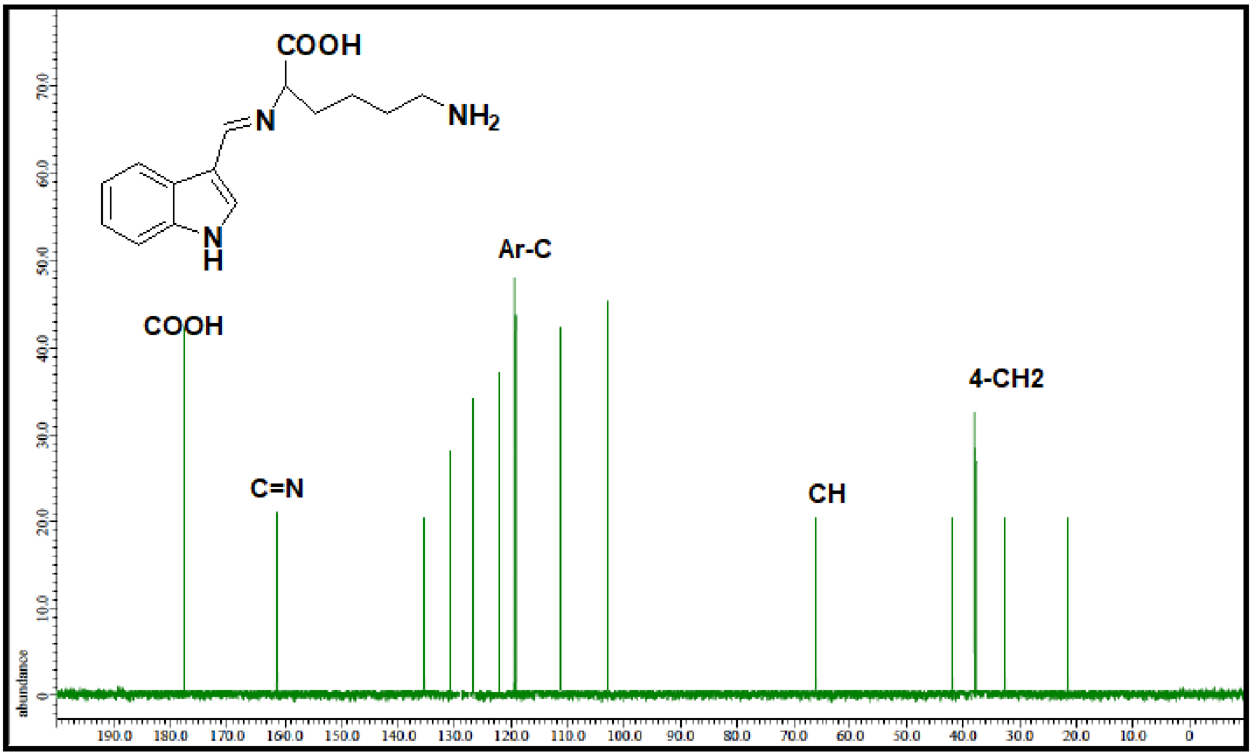

Figure S32. ^{13}C-NMR of compound 18

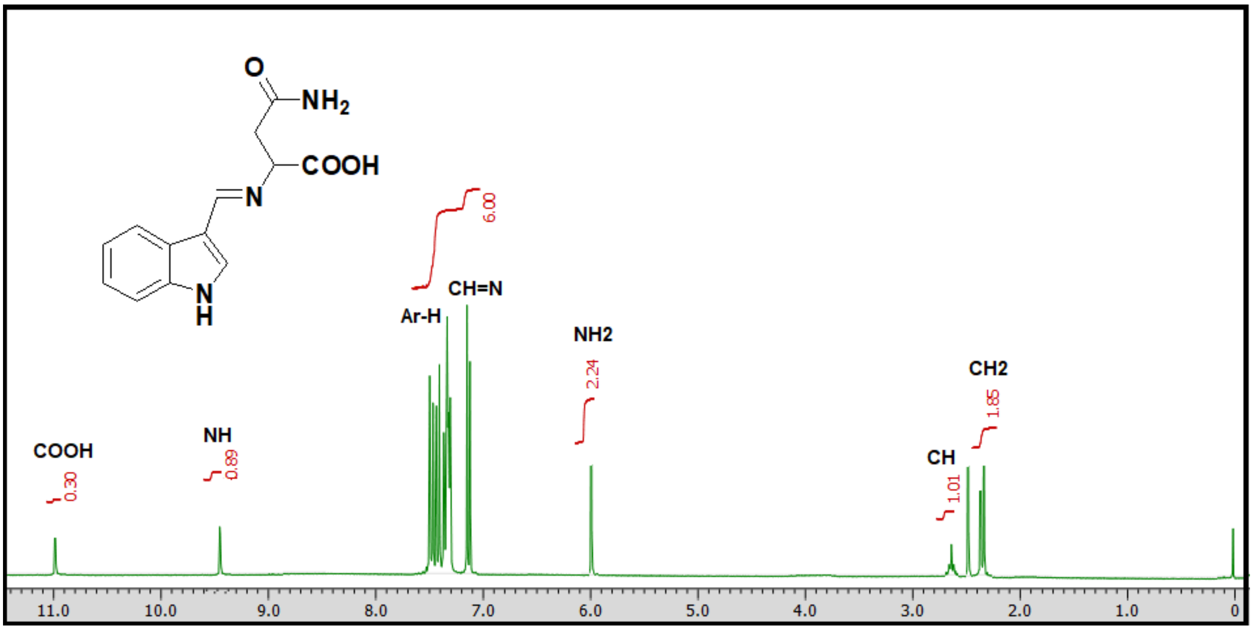

Figure S33. ^{1}H-NMR of compound 19

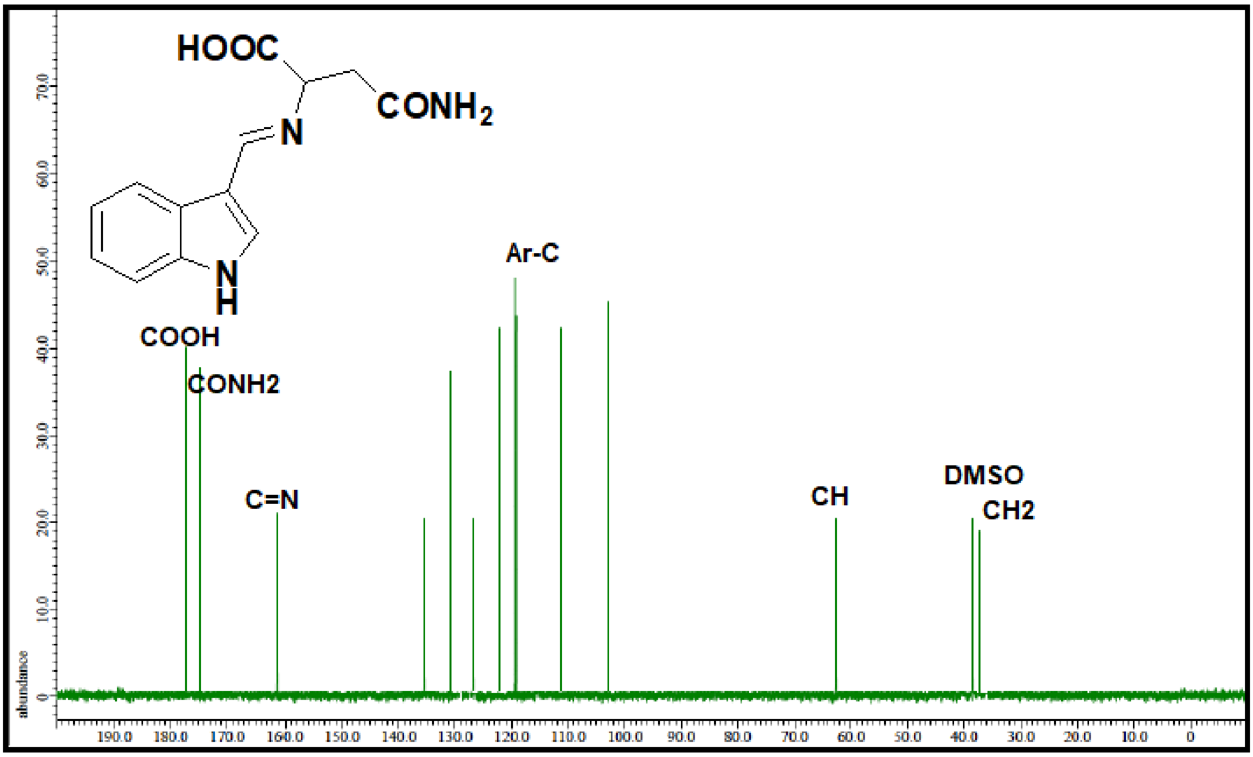

Figure S34. ^{13}C-NMR of compound 19

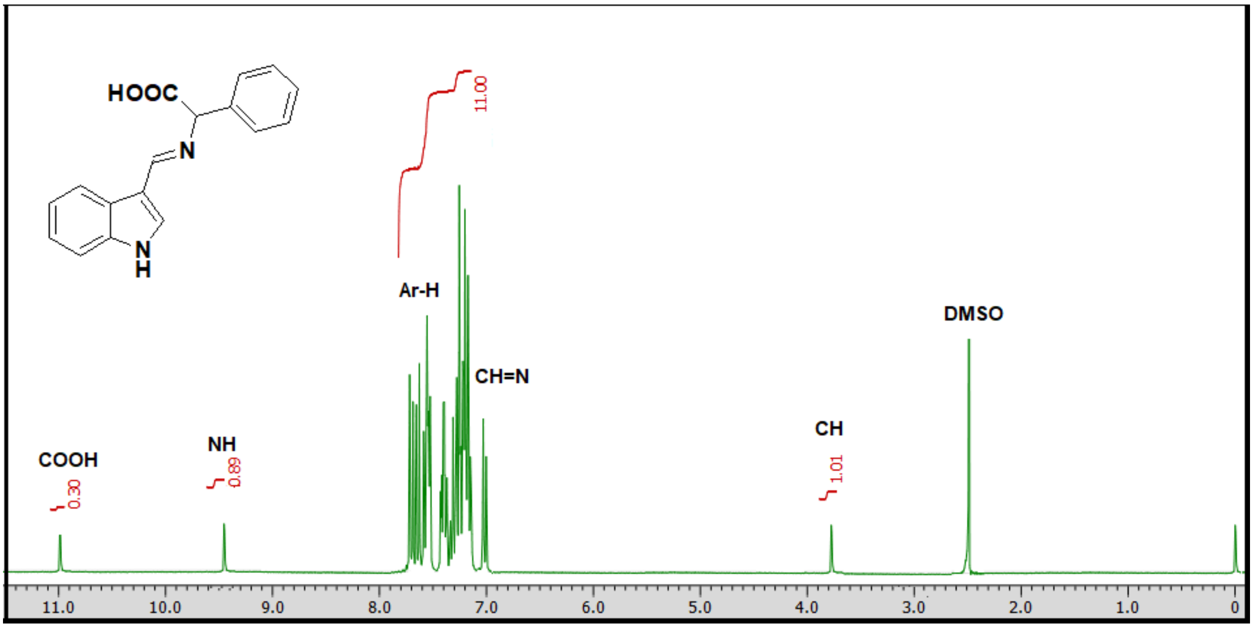

Figure S35. ^{1}H-NMR of compound 20.

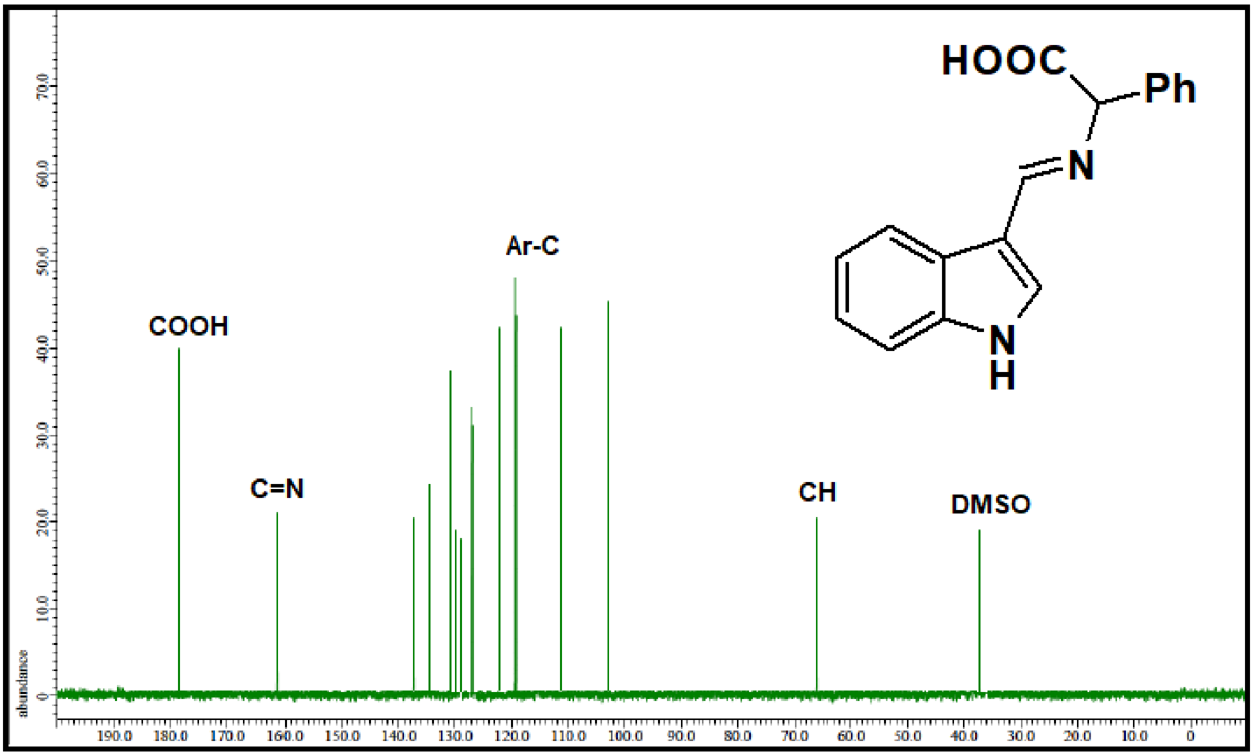

Figure S36. ^{13}C-NMR of compound 20

Compound 3-HK complex

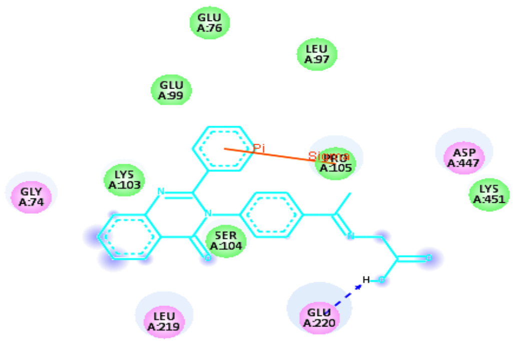

Compound 4-HK complex

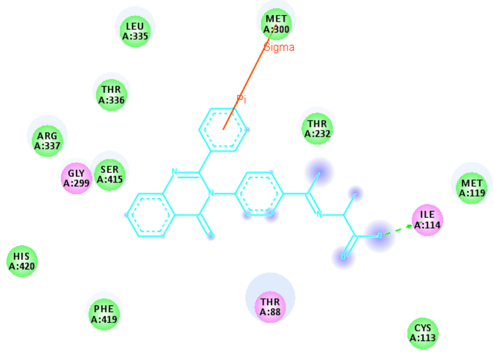

Copyright: © 2021 by the authors. Licensee MDPI, Basel, Switzerland. This article is an open access article distributed under the terms and conditions of the Creative Commons Attribution (CC BY) license (http://creativecommons.org/licenses/by/4.0/).

Compound 5-HK complex

Compound 6-HK complex

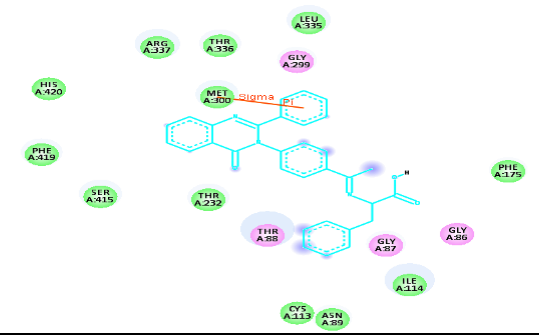

Compound 7-HK complex

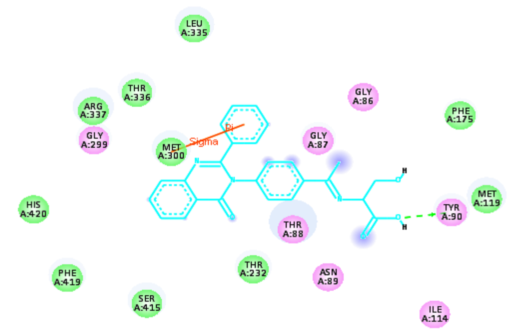

Compound 8-HK complex

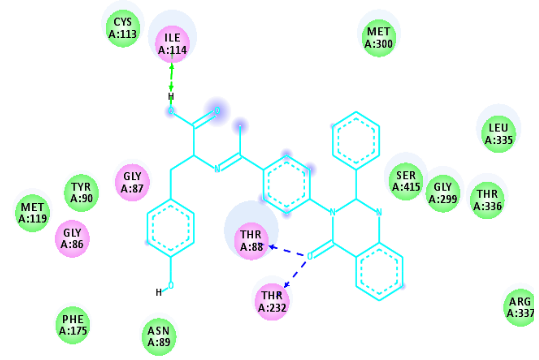

Compound 9-HK complex

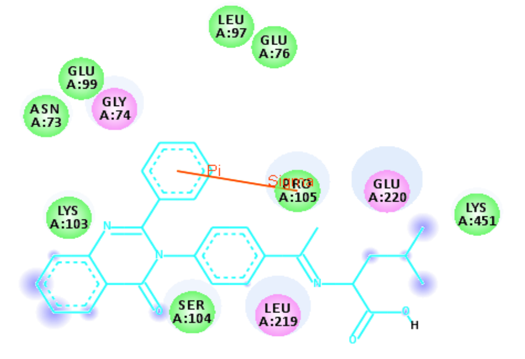

Compound 10-HK complex

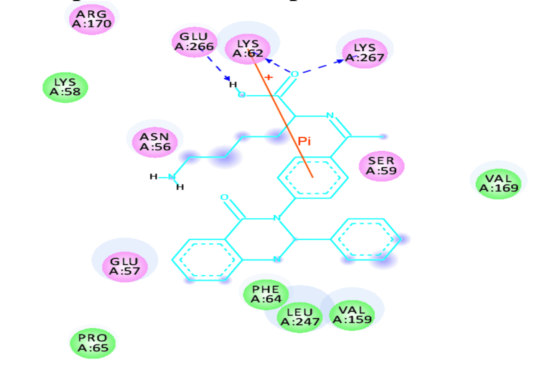

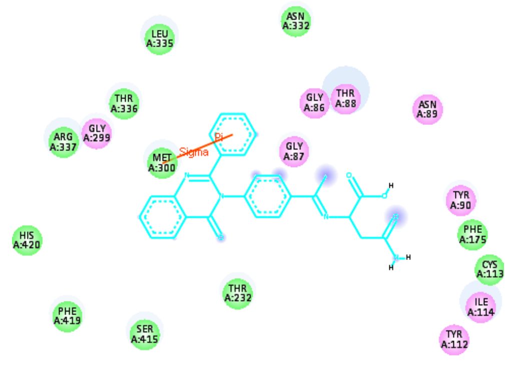

Compound 12-HK complex

Compound 13-HK complex

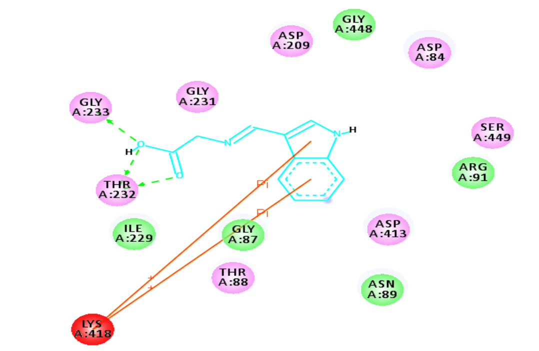

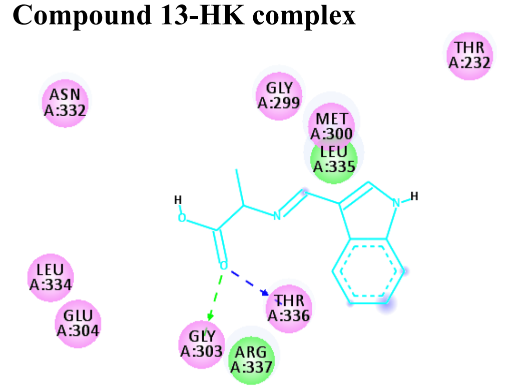

Compound 14-HK complex

Compound 15-HK complex

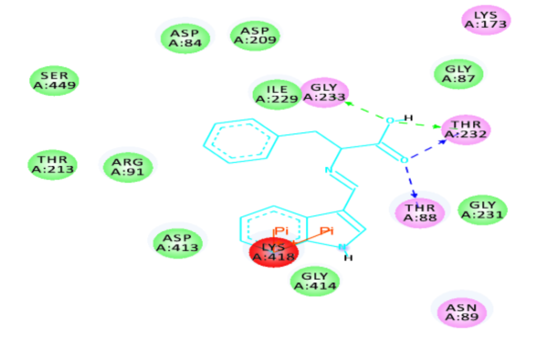

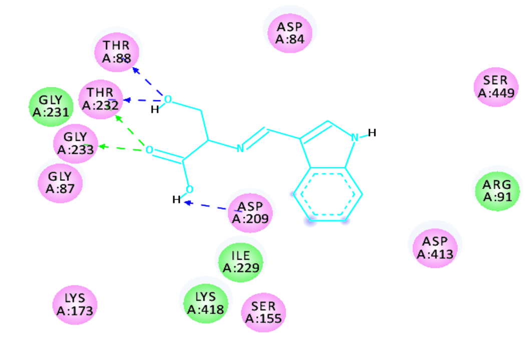

Compound 16-HK complex

Compound 17-HK complex

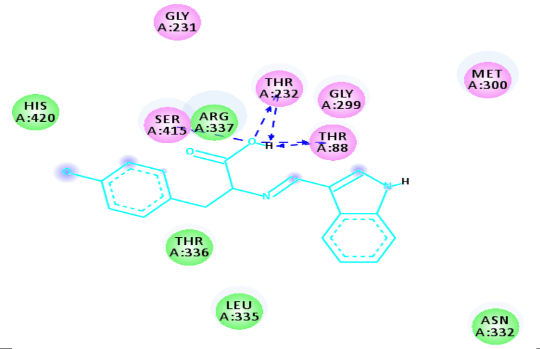

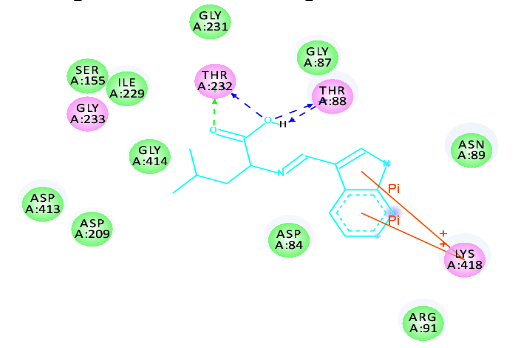

Compound 18-HK complex

Compound 19-HK complex

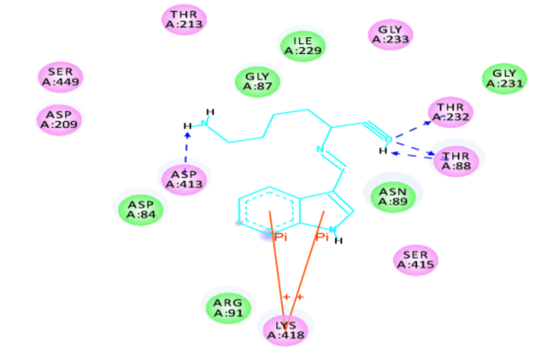

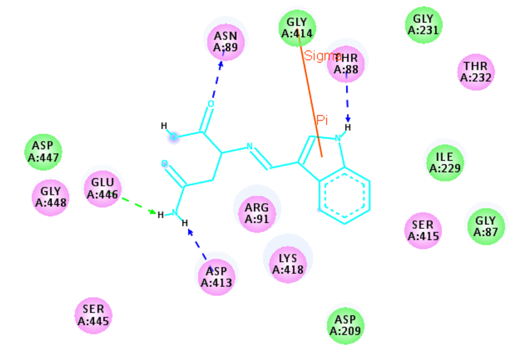

Figure S37. 2D of the other ligand-receptor interactions (first library). The ligand is shown is cyan color. The amino acid residues are displayed in 3 letter code. HBs are represented in green and blue dotted lines, while π- stacking interactions are in orange line color.

Compound 3- NADH complex

Compound 4- NADH complex

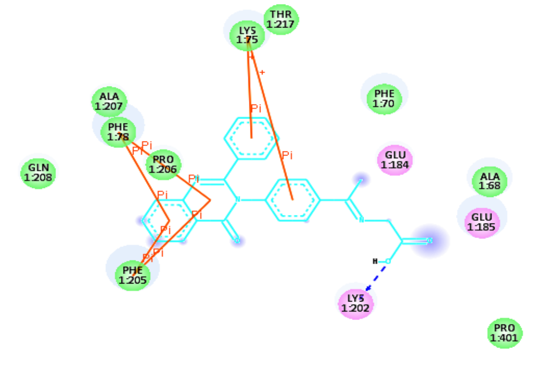

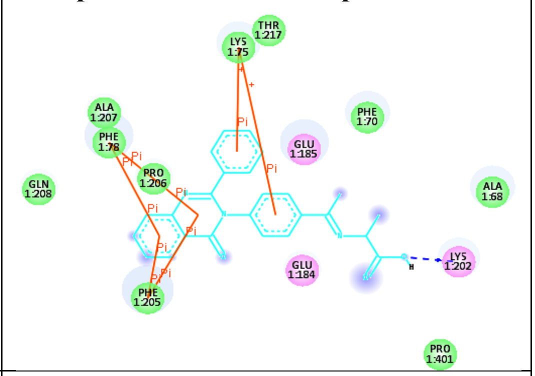

Compound 5- NADH complex

Compound 6- NADH complex

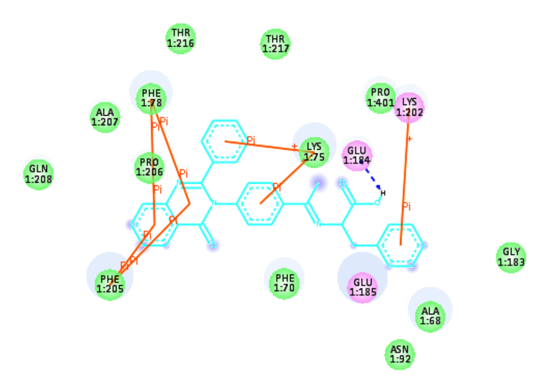

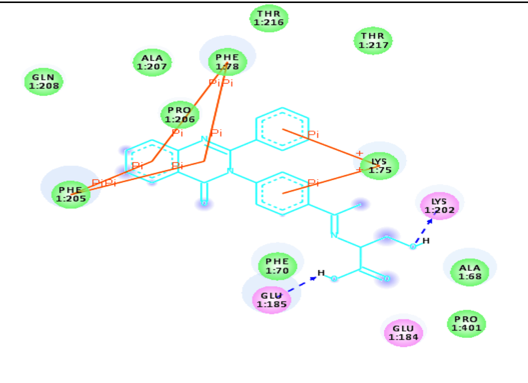

Compound 7- NADH complex **Compound 8- NADH complex**

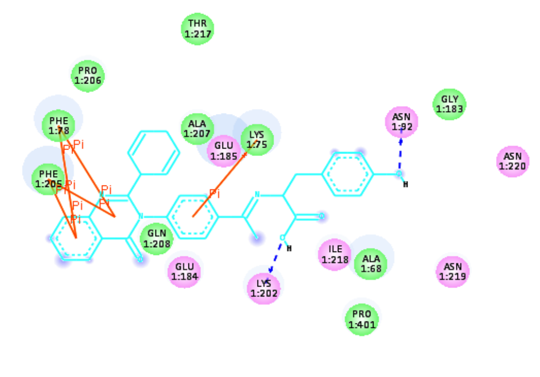

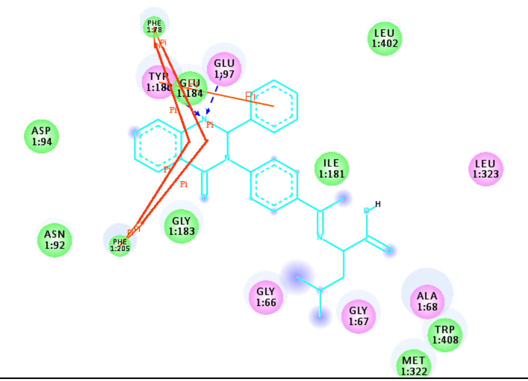

Compound 9- NADH complex **Compound 10- NADH complex**

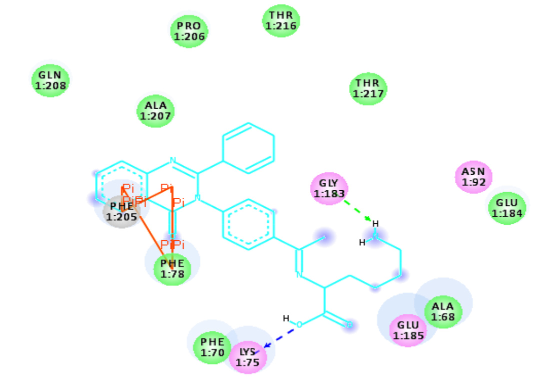

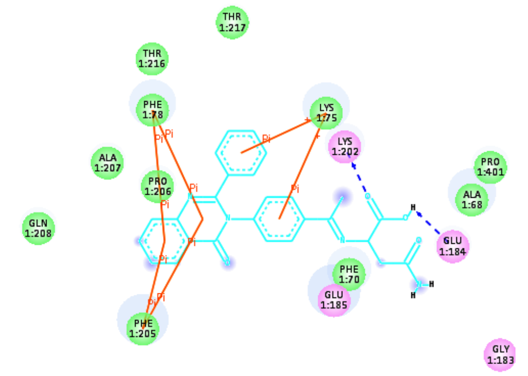

Compound 12- NADH complex **Compound 13- NADH complex**

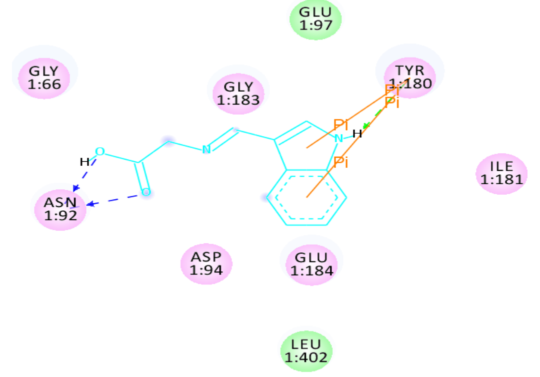

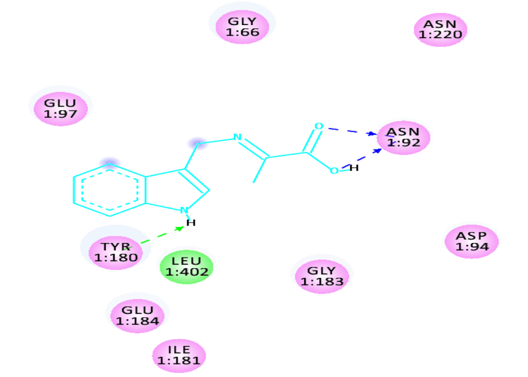

Compound 14- NADH complex **Compound 15- NADH complex**

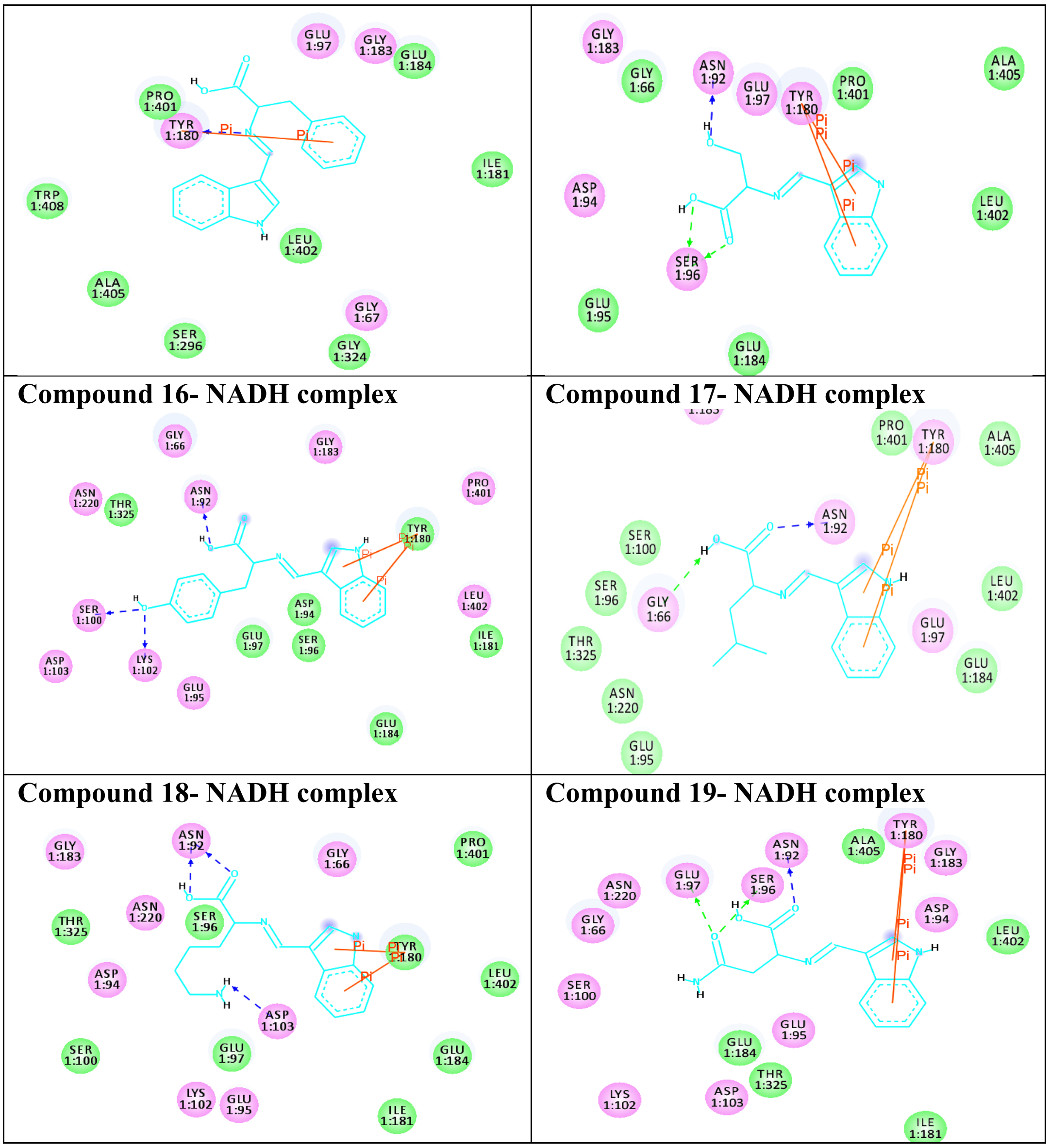

Figure S38. 2D of the other ligand-receptor interactions (second library). The ligand is shown is cyan color. The amino acid residues are displayed in 3 letter code. HBs are represented in green and blue dotted lines, while π- stacking are in orange line color.
